# Supplementary material for: Associations between introduction and withdrawal of a financial incentive and timing of attendance for antenatal care and incidence of small for gestational age: natural experimental evaluation using interrupted time series methods
Source: BMJ Open. 2018 Jan 31;8(1):e017697. doi: 10.1136/bmjopen-2017-017697 (PMC5829653; doi:10.1136/bmjopen-2017-017697)
Supplement: Author's manuscript [file bmjopen-2017-017697.draft_revisions.pdf]

BMJ Open

BMJ Open is committed to open peer review. As part of this commitment we make the peer review history of every article we publish publicly available.

When an article is published we post the peer reviewers' comments and the authors' responses online. We also post the versions of the paper that were used during peer review. These are the versions that the peer review comments apply to.

The versions of the paper that follow are the versions that were submitted during the peer review process. They are not the versions of record or the final published versions. They should not be cited or distributed as the published version of this manuscript.

BMJ Open is an open access journal and the full, final, typeset and author-corrected version of record of the manuscript is available on our site with no access controls, subscription charges or pay-per-view fees (http://bmjopen.bmj.com).

If you have any questions on BMJ Open's open peer review process please email editorial.bmjopen@bmj.com

BMJ Open

Effect of introduction and withdrawal of a financial incentive on timing of attendance for antenatal care and incidence of small for gestational age: natural experimental evaluation using interrupted time series methods

Journal:	*BMJ Open*
Manuscript ID	bmjopen-2017-017697
Article Type:	Research
Date Submitted by the Author:	11-May-2017
Complete List of Authors:	Adams, J; University of Cambridge, Centre for Diet and Activity Research, MRC Epidemiology Unit<br>van der Waal, Zelda; Newcastle University<br>Rushton, Steven; Newcastle University<br>Rankin, Judith; Newcastle University, Institute of Health & Society
**Primary Subject Heading**:	Public health
Secondary Subject Heading:	Obstetrics and gynaecology
Keywords:	Health policy < HEALTH SERVICES ADMINISTRATION & MANAGEMENT, PUBLIC HEALTH, OBSTETRICS, PREVENTIVE MEDICINE, Community child health < PAEDIATRICS

SCHOLARONE™
Manuscripts

Effect of introduction and withdrawal of a financial incentive on timing of attendance for antenatal care and incidence of small for gestational age: natural experimental evaluation using interrupted time series methods

Jean Adams, PhD;[1*] Zelda van der Waal, PhD;[2] Steve Rushton, PhD;[2] Judith Rankin, PhD[3]

[1]MRC Epidemiology Unit, University of Cambridge School of Clinical Medicine, Box 285 Institute of Metabolic Science, Cambridge Biomedical Campus, Cambridge CB2 0QQ, UK

[2]School of Biology, Ridley Building 2, Newcastle University, Claremont Road, Newcastle upon Tyne, NE1 7RU, UK

[3]Institute of Health & Society, Baddiley Clark Building, Newcastle University, Richardson Road, Newcastle upon Tyne, NE2 4AX, UK

*author for correspondence: jma79@medschl.cam.ac.uk; tel +44 1223 769 142

Keywords: reward, incentive, maternal, antenatal, prenatal, natural experiment

For peer review only

Abstract

Objectives

To determine whether introduction or withdrawal of a government-provided incentive of £190 ($235; €211) made to UK pregnant women who attended antenatal care by the 25th week of pregnancy was associated with changes in timing of first attendance for antenatal care, or incidence of small for gestational age.

Design

A natural experimental evaluation using an interrupted time series design.

Setting

One hospital-based maternity unit in the north of England.

Participants

34,589 women (and their live-born babies) who delivered at the study hospital and were known to have completed the 25th week of pregnancy in the 75 months before (January 2003–March 2009) introduction of the incentives, 21 months during (April 2009–December 2010) availability of the incentives, and 36 months after (January 2011–December 2013) withdrawal of the incentive.

Intervention

The Health in Pregnancy Grant was a maternal financial incentive of £190 ($235; €211) payable to pregnant women in the UK from the 25th week of pregnancy, contingent on them receiving routine antenatal care.

Primary and secondary outcome measures

The primary outcome was mean gestational age at booking. Secondary outcomes were proportion of women booking by 10, 18 and 25 weeks gestation; and proportion of babies that were small for gestational age.

Results

For peer review only

By 21 months after introduction of the grant (i·e. immediately prior to withdrawal), compared to what was predicted given prior trends, there was an reduction in mean gestational age at first antenatal care of 4·8 days (95% confidence intervals: 2·3 to 8·2). The comparable figure for 24 months after withdrawal was an increase of 14·0 days (95%CI: 2·8 to 16·8). No changes in incidence of small for gestational age babies were seen.

Conclusions

Financial incentives can improve timing of first antenatal care attendance; effects do not translate into changes in incidence of small for gestational age.

For peer review only

Article summary

Strengths and limitations of this study

- We used interrupted time series methods to evaluate this natural experiment; one of the strongest quasi-experimental research designs available.
- By including substantial data before and after interventions, we took account of underlying secular trends.
- However, interrupted time series designs are observational and we cannot categorically ascribe the changes documented to the intervention.
- Our primary outcomes was proportion of babies born small for gestational age - a substantial improvement on previous studies that use a simple low birth weight cut-off.
- Differences between women included and excluded from the analyses may limit external validity, as may our use of data from only one hospital.

For peer review only

Introduction

Financial incentives are increasingly used to encourage health promoting behaviours. However, few large, pragmatic, evaluations in high-income countries have been conducted.[1 2]

The Health in Pregnancy Grant (HiPG) was introduced in April 2009 as a one-off payment of £190 ($235; €211) payable to pregnant women, normally resident in the UK, after the 25th week of pregnancy, but before delivery. Women submitted a claim form, signed by their doctor or midwife confirming their expected delivery date and that they had received usual antenatal care.[3] A key aim of the HiPG was to act as an "incentive to seek the recommended health advice at the appropriate time".[3] Following a general election in 2010, the HiPG was withdrawn with women only able to claim if they reached the end of the 25th week of pregnancy before 1 January 2011.

England compares poorly to other European countries on perinatal outcomes.[4] One possible reason is poor attendance at antenatal care, which is associated with increased risk of small for gestational age (SGA),[5 6] and a range of adverse outcomes.[7-9] National guidance recommends that the first antenatal (or 'booking') visit should ideally take place by 10 weeks gestation and, at the latest, by 18 weeks.[10] Women living in more deprived circumstances tend to book later in pregnancy'.[11]

Health promoting financial incentives may be more effective in promoting one-off behaviours than complex behaviour change.[12-15] Antenatal care is a series of one-off behaviours and may be particularly responsive to incentives. However, a recent systematic review found only five trials of maternal incentives for antenatal care – three conducted in the USA and one each in Mexico and Honduras.[16] No effect on timing of antenatal care was found (although only one study investigated this).[17] No studies included birthweight or SGA as outcomes. A further observational study from the USA found no effect of an incentive on incidence of low birth weight.[18] One recent evaluation of the HiPG in Scotland reported no effect on birth weight, but a positive effect on the proportion of women booking by 25 weeks (other aspects of timing of attendance were not studied).[19]

For peer review only - http://bmjopen.bmj.com/site/about/guidelines.xhtml

More deprived people may be more responsive to financial incentives.[20 21] Other personal characteristics, such as age and previous experience of the behaviour incentivised, may also influence responsiveness. However, differential responses to health promoting financial incentives have not been systematically studied.[1]

The introduction and withdrawal of the HiPG provided a unique opportunity for a large-scale, pragmatic, natural experimental evaluation of a health promoting financial incentive.[22] Our research questions were: was the introduction or withdrawal of the HiPG associated with a change in the timing of booking, or incidence of SGA? Did any effect of the HiPG vary according to maternal age, parity or deprivation?

Methods

We used an interrupted time series (ITS) design.

Data and inclusion criteria

We used routine data from a maternity unit in a tertiary hospital in northern England, extracted in May 2015. The study hospital is a general teaching hospital with over 1000 beds in a town with a population of ~175,000 people. Both the town and surrounding areas are more deprived than the English average.

Participants were women (and their live-born babies) who delivered at the study hospital and were known to have completed the 25th week of pregnancy in the 75 months before (January 2003–March 2009) introduction of the HiPG, 21 months during (April 2009–December 2010) availability of the HiPG, and 36 months after (January 2011–December 2013) withdrawal of the HiPG. The time periods included were pragmatically arrived at based on when data was available from, and when the HiPG was introduced and withdrawn. Our final data set of 120 monthly data points, substantially exceeds the minimum requirements for ITS.[23] As calculation of when women reached the 25th week of pregnancy depended on knowing the date of their last menstrual period (LMP), women for whom this date was missing were excluded.

For peer review only - http://bmjopen.bmj.com/site/about/guidelines.xhtml

Women who had a termination or experienced a stillbirth were excluded, as were women with missing data on any variable of interest. Women who delivered more than one live baby in any one pregnancy, or had more than one pregnancy that resulted in a live birth during the study period, were included with each baby counted as a separate 'case'.

Outcome measures

The primary outcome was mean gestational age at booking, calculated from dates of booking (recorded by antenatal care staff) and LMP (self-reported). As national guidance recommends booking ideally before 10 weeks, and definitely before 18 weeks, and the HiPG was available to women from the 25th week, the proportion of women booking by 10, 18 and 25 weeks gestation were secondary outcomes.[10]

The final secondary outcome was proportion of SGA babies, i.e. birth weight z-score below the 10th percentile for sex-specific gestational age.[24] This was calculated using infant sex, birth weight and dates of LMP and delivery (all except LMP recorded by antenatal care and delivery staff).

Other variables of interest

We studied whether any effects of the HiPG on the outcomes varied according to maternal age at delivery (in years, calculated from maternal date of birth and date of delivery and divided into three groups: <25, 25-34, or 35+ years), parity (self-reported and considered as 0 or 1+ in analyses) and socio-economic position. Socio-economic position was measured using the Index of Multiple Deprivation (IMD) 2007 rank assigned to maternal address at delivery.[25] IMD ranks were divided into thirds for analysis based on the distribution across England.

Data preparation

Data cleaning aimed to exclude data that were implausible. Date of LMP was recorded as month and year only in around 20% of cases. To include these cases, day of month was set to the 1st. Gestational age at first antenatal care of less than 28 days (4 weeks) or more than 308 days (44 weeks), gestational age at delivery

For peer review only - http://bmjopen.bmj.com/site/about/guidelines.xhtml

124 of less than 24 weeks and more than 44 weeks, or birth weight z-scores of less than -3 or more than 3 were
125 recoded as missing.

126 *Data analyses*

127 An uncontrolled, multiple time points, ITS design was used. The unit of analysis was the month in which
128 women entered the 25th week of pregnancy. ITS models estimate the change in 'level' and 'trend' of the
129 outcome of interest associated with the intervention. The change in level is the difference in intercepts
130 between regression lines estimated from observations before and after the intervention. The change in
131 trend is the difference in slopes. In the case of two 'interventions' (e.g. introduction and withdrawal of the
132 HiPG), two changes in level and trend are estimated.

133 General linear models were used allowing for autoregressive and moving average correlation structures as
134 appropriate. These allow any effect of seasonality to be taken into account. Firstly, associations between
135 introduction and withdrawal of the HiPG and the outcomes of interest were assessed in the whole cohort,
136 using separate models for each outcome. Final models were used to calculate estimated absolute and
137 relative effects on each outcome of the introduction of the HiPG at 21 months post-implementation
138 (immediately prior to withdrawal), and 24 months after withdrawal, with 95% confidence intervals.[26]
139 Interaction terms were then used to determine whether the effects of the introduction or withdrawal of the
140 HiPG varied by maternal age group, parity or IMD tertile.

141 Data preparation was conducted in StataSE v14; data analysis in R v3.3.1 and RStudio v0.99.903.

142 **Results**

143 *Sample description*

144 Of 39,571 women who delivered at the study hospital and were known to have reached the 25th week of
145 gestation between 1st January 2003 and 31st December 2013, full data were available for 34,589 (87.4%).
146 Characteristics of those for whom full data was and was not available during each study stage are described

For peer review only - http://bmjopen.bmj.com/site/about/guidelines.xhtml

in Table 1. Most exclusions were due to missing information on birth weight. Typically, women included in the analyses were aged 25-34 years, of parity 1 or more, lived in the most deprived third of areas in England, and booked by 10 weeks gestation. Women excluded from the analyses tended to be younger, live in more deprived areas, and booked later in their pregnancies than women included. Similar differences between women included and excluded from the analyses were seen in each of the three study periods.

Sample-wide effects of the intervention

Final models for each outcome are summarised in Table 2 and plotted in Figure 1. Introduction of the HiPG was associated with an immediate increase in mean gestational age at booking, and a decrease in the proportion booking by 18 and 25 weeks. That is, the immediate effect was for these outcomes to get clinically 'worse'. However, introduction of the HiPG was also associated with an improvement in the trend in mean gestational age at booking and proportion booking by 10, 18 and 25 weeks. That is, the longer term effect was a change in trend of these outcomes towards greater clinical improvement over time.

Withdrawal of the HiPG was not associated with any level changes in outcomes. However, it was associated with a change in trend in mean gestational age at booking and proportion booking by 18 and 25 weeks towards less clinical improvement over time. The introduction or withdrawal of the HiPG was not associated with any changes in the level or trend in the proportion of babies who were SGA.

Table 3 shows the absolute and relative impact of the introduction and withdrawal of the HiPG on each outcome at 21 months post introduction and 24 months post withdrawal. By 21 months after introduction of the HiPG, compared to the counterfactual of what was predicted given trends prior to the introduction of the HiPG, there was a reduction in mean gestational age at booking of 4.8 days (95% confidence intervals: 2.3 to 8.2), an increase in the proportion of women booking by 18 weeks of 2.2% (95%CI: 1.2 to 3.9), and an increase in the proportion of women booking by 25 weeks of 1.9% (95%CI: 0.6 to 3.5). Compared to the counterfactual of what was predicted to occur given trends when the HiPG was available, by 24 months after withdrawal, there was an increase in mean gestational age at booking of 14.0 days (95% CI: 2.8 to 16.8), a

For peer review only - http://bmjopen.bmj.com/site/about/guidelines.xhtml

decrease in the proportion of women booking by 18 weeks of 7.6% (95% CI: 2.2 to 7.9), and a decrease in the proportion of women booking by 25 weeks of 8.3% (95% CI: 3.1 to 8.6).

Differential effects of the intervention across population sub-groups

Models including interaction terms for maternal age, parity and IMD tertile are summarised in Tables 4-6. There were no interactions with parity. The effect of introduction and withdrawal of the HiPG on trend in mean gestational age at booking varied by age group (Figure 2), with greater effects in older women.

The effect of introduction of the HiPG on mean gestational age at booking and proportion booking by 18 and 25 weeks varied by IMD group (Figure 3). The introduction of the HiPG was associated with a progressively larger level change (towards older gestational age at booking, and lower proportion booking by 18 or 25 weeks) as deprivation decreased.

Discussion

Statement of principal findings

This is the first evaluation of the HiPG in England, the first evaluation of a financial incentive for attendance at antenatal care on incidence of SGA, and one of the largest pragmatic evaluations of a health promoting financial incentive in a high-income country. Introduction of the HiPG was associated with immediate deteriorations in timing of booking, but longer term improvements over time. By 21 months after introduction of the HiPG (immediately prior to its withdrawal), mean gestational age at booking had decreased by 4.8 days compared to what would have been expected had it not been introduced. Withdrawal of the HiPG was not associated with any immediate changes in timing of booking, but it was associated with longer-term deteriorations in timing of booking over time. By 24 months after withdrawal, mean gestational age at booking had increased by 14.0 days compared to what would have been expected had it not been withdrawn. No effects of the HiPG on the incidence of SGA were found. The effect of the HiPG did not vary by parity. The positive effects of the introduction of the HiPG on trends in gestational age at booking were

For peer review only

194 greater in older women. The immediate negative effect of the introduction of the HiPG on timing of booking was more pronounced in less deprived groups.

196 *Strengths and weaknesses of methods*

197 The ITS approach is one of the strongest quasi-experimental research designs.[23 27] By including substantial data before and after interventions, we took account of underlying secular trends. By studying outcomes at the population-, rather than individual-, level confounding by individual-level variables was avoided. Our large data set with 120 monthly data points, substantially exceeds the minimum requirements for ITS.[23] By including auto-regressive and moving-average functions, any biases introduced by the serial nature of the data (including seasonality) were accounted for. However, ITS designs are observational and we cannot categorically ascribe the changes documented to the HiPG. Whilst we are not aware of any co-interventions likely to have influenced the outcomes concurrent with the HiPG, it is difficult to absolutely exclude these.

205 A major strength of our study is the use of SGA. Unlike a simple cut-off for low birth weight, SGA allows sex and gestational age differences in birth weight to be taken into account.

207 The data we used is likely to contain recording, reporting and transcription errors. Some of these may have introduced bias. 'Feasibility' limits were used for some variables and may have led to misclassification.

209 Cases included in the analytical cohort differed from those excluded. However, as differences between women included and excluded from the analyses were similar in all three study periods, this is unlikely to introduce bias and so we did impute missing data. Differences between women included and excluded from the analyses may limit external validity, as may our use of data from only one hospital.

213 *Interpretation of findings*

214 Our finding that the introduction of the HiPG was associated with an immediate deterioration in timing of booking is not immediately explainable. It may reflect an implementation phase, where the process for obtaining the HiPG was not yet fully understood. Whilst we could have conducted further analyses excluding

For peer review only - http://bmjopen.bmj.com/site/about/guidelines.xhtml

an implementation period (e.g. four months after introduction of the HiPG), this would have been *post-hoc* justified.

The longer-term associations of the introduction of the HiPG on markers of timing at booking are in line with the intention of the intervention – that women should attend for antenatal care earlier in their pregnancies. One previous study found no effect on timing of first attendance of providing a voucher for a taxi journey to the antenatal clinic,[17] whilst a further evaluation of the HiPG found it was associated with a positive effect on the proportion of women booking by 25 weeks that disappeared after withdrawal.[19] The substantial difference in incentive value of the HiPG, compared to previous incentives, may explain these differences.

The finding that withdrawal of the HiPG was associated with deterioration of the benefits of its introduction on timing of booking is also not unexpected. On the whole, different women would have been pregnant when the HiPG was and was not available, meaning sustained effects would be highly unlikely.

Changes in timing of attendance for antenatal care associated with the introduction and withdrawal of the HiPG did not translate into differences in the proportion of SGA babies. This may be because the effect size (of 4.8 days at 21 months) was too small to impact on SGA. Two previous studies that examined the effect of incentives for antenatal care on incidence of low birth weight (rather than SGA) also reported no effect.[18 19]

Although the HiPG was only available from the 25th week of pregnancy, we found that its introduction was associated with changes in the proportion of women booking by both 10 and 18 weeks. This indicates that the impact of health promoting financial incentives may not be as specific as previously thought.[28] The HiPG may have been associated with a larger effect on timing of booking if it had been contingent on booking earlier in pregnancy.

We did not find any evidence that the associations of the introduction or withdrawal of the HiPG with the outcomes studied varied by parity. This suggests that prior experience of antenatal care did not diminish the impact of the HiPG. However, the association of the introduction of the HiPG with improvements in gestational age at booking over time were greater in older women. This suggests that age may be a

For peer review only - http://bmjopen.bmj.com/site/about/guidelines.xhtml

determinant of responsiveness to financial incentives in this context. Our data are consistent with the suggestion that those with fewer resources are particularly responsive to incentives.[29] Whilst introduction of the HiPG was associated with an immediate negative change in timing of first antenatal care, this was least pronounced in women living in the most deprived areas.

Implications of findings for policy, practice and research

It is possible that larger incentives, contingent on attendance earlier than 25 weeks, may have greater impacts on timing of antenatal care and clinical outcomes than seen here. Future research could explore how effects on antenatal care attendance vary with incentive value and timing.

As we used routine data in a retrospective analysis conducted more than two years after withdrawal of the HiPG, we were unable to explore how women and other stakeholders responded to the HiPG. In particular, we do not know what women spent the HiPG on, how doctors and midwives discussed it with women, or how appropriate stakeholders thought it was. These factors may have influenced effectiveness and variations in effectiveness between sub-groups.[30]

Conclusions

The introduction of the HiPG was associated with an improvement in timing of first attendance for antenatal care of almost five days by 21 months post-implementation. Withdrawal of the grant diminished this trend towards greater improvement over time. Neither the introduction nor withdrawal of the HiPG was associated with a change in proportion of babies who were SGA. Effects did not vary by parity. Introduction of the HiPG was associated with greater long-term benefits on timing at booking in older women. Those living in more deprived circumstances showing the most positive initial response.

For peer review only

Funding

This work is produced under the terms of a Career Development Fellowship research training fellowship issued by the National Institute of Health Research [grant number CDF-2011-04-001] to JA. The views expressed are those of the authors and not necessarily those of the NHS, The National Institute for Health Research or the Department of Health. JA is supported by the Centre for Diet and Activity Research (CEDAR), a UKCRC Public Health Research Centre of Excellence. Funding from the British Heart Foundation, Cancer Research UK, Economic and Social Research Council, Medical Research Council, the National Institute for Health Research, and the Wellcome Trust, under the auspices of the UK Clinical Research Collaboration, is gratefully acknowledged [grant number MR/K023187/1].

Role of the funding sources

The funders played no role in any aspect of study design, data analysis, writing or the decision to submit. All authors had full access to all the data and had final responsibility for the decision to submit. No authors were paid by a pharmaceutical company or other agency to write this article. All authors are independent from the funders.

Competing interests

All authors have completed the ICMJE uniform disclosure form at www.icmje.org/coi_disclosure.pdf and declare: JA had financial support from the National Institute for Health Research and the Centre for Diet and Activity Research (as described under ‘funding’) for the submitted work; no financial relationships with any organisations that might have an interest in the submitted work in the previous three years; no other relationships or activities that could appear to have influenced the submitted work.

Contributions

JA conducted the literature searches, obtained the data, conducted the data analysis and led writing. ZvdW, SR, and JR contributed to study design, development of the analysis plan, interpretation of the data and critically reviewed previous versions of the final manuscript. JA will act as guarantor.

For peer review only

Research ethics

Ethics approval was granted by the East of England Norfolk NHS Research Ethics Committee (12/EE/0386). The routine hospital data used in this study was anonymised before transfer to the research team and the ethics committee determined that explicit patient consent was not required.

Data Sharing

No additional data available.

For peer review only

References

1. Giles E, Robalino S, McColl E, et al. Systematic review, meta-analysis and meta-regression of the effectiveness of financial incentives for encouraging healthy behaviours. *PLoS ONE* 2014;9 doi: 10.1371/journal.pone.0090347

2. Mantzari E, Vogt F, Shemilt I, et al. Personal financial incentives for changing habitual health-related behaviors: A systematic review and meta-analysis. *Prev Med* 2015;75:75-85. doi: 10.1016/j.ypmed.2015.03.001 [published Online First: 2015/04/07]

3. HM Revenue & Customs, The Royal College of Midwives. Health in Pregnancy Grant: Frequently Asked Questions 2009 [Available from: http://www.direct.gov.uk/en/groups/dg_digitalassets/@dg/@en/documents/digitalasset/dg_173401.pdf accessed 25 March 2010.

4. EURO-PERISTAT project, SCPE, EUROCAT, et al. European Perinatal Health Report. Paris, 2008.

5. VanderWeele TJ, Lantos JD, Siddique J, et al. A comparison of four prenatal care indices in birth outcome models: Comparable results for predicting small-for-gestational-age outcome but different results for preterm birth or infant mortality. *J Clin Epidemiol* 2009;62(4):438-45.

6. Heaman M, Newburn-Cook C, Green C, et al. Inadequate prenatal care and its association with adverse pregnancy outcomes: A comparison of indices. *BMC Pregnancy and Childbirth* 2008;8(1):15.

7. Lin C, Chen C, Chen P, et al. Risks and causes of mortality among low-birthweight infants in childhood and adolescence. *Paediatr Perinat Epidemiol* 2007;21(5):465-72.

8. Mikkola K, Ritari N, Tommiska V, et al. Neurodevelopmental outcome at 5 years of age of a national cohort of extremely low birth weight infants who were born in 1996-1997. *Pediatrics* 2005;116(6):1391-400. doi: 10.1542/peds.2005-0171 [published Online First: 2005/12/03]

9. Tommiska V, Heinonen K, Ikonen S, et al. A national short-term follow-Up study of extremely low birth weight infants born in Finland in 1996-1997. *Pediatrics* 2001;107(1):E2. [published Online First: 2001/01/03]

10. National Collaborating Centre for Women's and Children's Health. Antenatal care: Routine care for the healthy pregnant woman. London: National Institute for Health and Clinical Excellence, 2008.

11. Rowe RE, Garcia J. Social class, ethnicity and attendance for antenatal care in the United Kingdom: a systematic review. *J Public Health* 2003;25(2):113-19. doi: 10.1093/pubmed/fdg025

12. Sutherland K, Christianson JB, Leatherman S. Impact of Targeted Financial Incentives on Personal Health Behavior: A Review of the Literature. *Med Care Res Rev* 2008;65(6_suppl):36S-78. doi: 10.1177/1077558708324235

13. Marteau T, Ashcroft R, Oliver A. Using financial incentives to achieve healthy behaviour. *BMJ* 2009;338 doi: 10.1136/bmj.b1415

14. Jochelson K. Paying the Patient: improving health using financial incentives. London: King's Fund, 2007.

15. Forde I, Zeuner D. Financial incentives to promote social mobility. *BMJ* 2009;339:b3219. doi: 10.1136/bmj.b3219

For peer review only

16. Till SR, Everetts D, Haas DM. Incentives for increasing prenatal care use by women in order to improve maternal and neonatal outcomes. *Cochrane Database Syst Rev* 2015(12) doi: 10.1002/14651858.CD009916.pub2

17. Melnikow J, Paliescheskey M, Stewart G. Effect of a transportation incentive on compliance with the first prenatal appointment: a randomized trial. *Obstet Gynecol* 1997;89:1023-7.

18. Rosenthal M, Li Z, Robertson A, et al. Impact of financial incentive for prenatal care on birth outcomes and spending. *HSR: Health Services Research* 2009;44:1465-79.

19. Leyland A, Ouedraogo S, Gray R, et al. Evaluating health in pregnancy grants in Scotland: a natural experiment. *J Epidemiol Community Health* 2016;70(S1):A47-8.

20. Lorenc T, Petticrew M, Welch V, et al. What types of interventions generate inequalities? Evidence from systematic reviews. *J Epidemiol Community Health* 2012;67(2):190-3. doi: 10.1136/jech-2012-201257

21. White M, Adams J, Heywood P. How and why do interventions that increase health overall widen inequalities within populations? In: Babones S, ed. Social inequality and public health. Bristol: Policy Press 2009:65-82.

22. Craig P, Cooper C, Gunnell D, et al. Using natural experiments to evaluate population health interventions: guidance for producers and users of evidence: Medical Research Council, 2011.

23. Jandoc R, Burden AM, Mamdani M, et al. Interrupted time series analysis in drug utilization research is increasing: systematic review and recommendations. *J Clin Epidemiol* 2015;68(8):950-6. doi: 10.1016/j.jclinepi.2014.12.018 [published Online First: 2015/04/22]

24. Bonellie S, Chalmers J, Gray R, et al. Centile charts for birthweight for gestational age for Scottish singleton births. *BMC Pregnancy & Childbirth* 2008;8:5. doi: 10.1186/1471-2393-8-5 [published Online First: 2008/02/27]

25. Department of Communities and Local Government. The English Indices of Deprivation 2010 2011 [Available from: http://www.communities.gov.uk/publications/corporate/statistics/indices201015 October 2012.

26. Zhang F, Wagner AK, Soumerai SB, et al. Methods for estimating confidence intervals in interrupted time series analyses of health interventions. *J Clin Epidemiol* 2009;62(2):10.1016/j.jclinepi.2008.08.007. doi: 10.1016/j.jclinepi.2008.08.007

27. Penfold RB, Zhang F. Use of interrupted time series analysis in evaluating health care quality improvements. *Academic Pediatrics* 2013;13(6 Suppl):S38-44. doi: 10.1016/j.acap.2013.08.002 [published Online First: 2013/12/07]

28. Adams J, Giles E, McColl E, et al. Carrots, sticks, and health behaviours: a framework for documenting the complexity of financial incentive interventions to change health behaviours. *Health Psychology Review* 2014;8(3):286-95.

29. Giles E, Robalino S, Sniehotta F, et al. Acceptability of financial incentives for encouraging uptake of healthy behaviours: A critical review using systematic methods. *Prev Med* 2015;73 doi: 10.1016/j.ypmed.2014.12.029

30. Giles EL, Adams JM. Capturing public opinion on public health topics: a comparison of experiences from a systematic review, focus group study, and analysis of online, user-generated content. *Frontiers in Public Health* 2015;3 doi: 10.3389/fpubh.2015.00200

For peer review only

Table 1. Characteristics of those included and excluded from the analytical cohort

		Before HiPG availability (January 2003 – March 2009)		During HiPG availability (April 2009 – December 2010)		After HiPG availability (January 2011 – December 2013)		Full study period (January 2003 – December 2013)	
Variable	Level	Included[1]	Excluded[2]	Included[1]	Excluded[2]	Included[1]	Excluded[2]	Included[1]	Excluded[2]
N(%)		18 744	2816	6126	862	9719	1304	34 589	4982
Maternal age, n(%)	<25 years	6359 (33.9)	1327 (47.1)	2039 (33.3)	374 (43.4)	2836 (29.2)	523 (40.1)	11 234 (32.5)	2224 (44.6)
	25-34 years	9684 (51.7)	1208 (42.9)	3272 (53.4)	405 (47.0)	5577 (57.4)	651 (49.9)	18 533 (53.6)	2264 (45.4)
	35 years +	2701 (14.4)	281 (10.0)	815 (13.3)	83 (9.6)	1306 (13.4)	130 (10.0)	4822 (13.9)	494 (9.9)
	Data not available, n(%)	0	0[3]	0	0[3]	0	0[3]	0	0[3]
Parity, n(%)	0	8077 (43.1)	1288 (45.7)	2670 (43.6)	378 (43.9)	3944 (40.6)	585 (44.9)	14 691 (42.5)	2251 (45.2)
	1+	10 667 (56.9)	1528 (54.3)	3456 (56.4)	484 (56.2)	5775 (59.4)	719 (55.1)	19 898 (57.5)	2731 (54.8)
	Data not available, n(%)	0	0[3]	0	0	0	0[3]	0	0[3]
Index of multiple deprivation group, n(%)	Most deprived	10 820 (57.7)	1777 (63.1)	3566 (58.2)	547 (63.5)	5821 (59.9)	794 (60.9)	20 207 (58.4)	3118 (62.6)
	Moderately deprived	4213 (22.5)	490 (17.4)	1330 (21.7)	160 (18.6)	2230 (22.9)	220 (16.9)	7773 (22.5)	870 (17.5)
	Least deprived	3711 (19.8)	361 (12.8)	1230 (20.1)	92 (10.7)	1668 (17.2)	128 (9.8)	6609 (19.1)	581 (11.7)
	Data not available, n(%)	0	188 (6.7)[3]	0	63 (7.3)[3]	0	162 (12.4)[3]	0	413 (8.3)[3]
Study outcomes	Mean (SD) gestational age at booking, days	76.7 (35.8)	106.2 (45.5)[4]	77.8 (41.3)	99.3 (39.8)[4]	71.8 (31.8)	95.0 (39.1)[4]	75.6 (35.8)	102.0 (43.2)[4]
	Booked by 10 weeks, n(%)	10 261 (54.7)	442 (17.0)[4]	3540 (57.8)	152 (18.8)[4]	6127 (63.0)	283 (23.9)[4]	19 928 (57.6)	877 (19.1)[4]
	Booked by 18 weeks, n(%)	17 579 (93.8)	2012 (77.7)[4]	5675 (92.6)	676 (83.7)[4]	9307 (95.8)	1022 (86.3)[4]	32 561 (94.1)	3719 (81.0)[4]
	Booked by 25 weeks, n(%)	18 098 (96.6)	2397 (92.1)[4]	5846 (95.4)	766 (94.8)	9477 (97.5)	1129 (95.4)[4]	33 421 (96.6)	4292 (93.4)[4]
	Small for gestational age, n(%)	2346 (12.5)	17 (8.3)	743 (12.1)	7 (11.5)	1163 (12.0)	25 (14.9)	4252 (12.3)	49 (11.3)
	Data not available on time at booking, n(%)	0	214 (7.6)	0	54 (6.3)	0	120 (9.2)	0	388 (7.8)
	Data not available on birth weight, n(%)	0	2611 (92.7)	0	801 (92.9)	0	1136 (87.1)	0	4548 (91.3)

For peer review only

For peer review only - http://bmjopen.bmj.com/site/about/guidelines.xhtml

[1]Women who delivered at the study hospital, were known to have reached the 25th week of gestation between 1st January 2003 and 31st December 2013 and had available data on all variables of interest; [2]Women who delivered at the study hospital, were known to have reached the 25th week of gestation between 1st January 2003 and 31st December 2013 and did not have available data on all variables of interest; [3]chi-squared test indicates difference in distribution of levels between included and excluded at a level of $p<0.01$; [4]t-test indicates difference in means between included and excluded at a level of $p<0.01$

HiPG: Health in Pregnancy Grant

For peer review only

For peer review only - http://bmjopen.bmj.com/site/about/guidelines.xhtml

Table 2. Summary of interrupted time series models of the effect of the introduction and withdrawal of the Health in Pregnancy Grant, coefficients (95% CI)

Model variable	Mean gestational age at booking (days)	Proportion booking by 10 weeks	Proportion booking by 18 weeks	Proportion booking by 25 weeks	Proportion of babies SGA
Time	0.02 (-0.01 to 0.05)	0.00[1] (-0.00 to 0.00)	**-0.00 (-0.00 to -0.00)[2]**	0.00 (-0.00 to 0.00)	0.00 (-0.00 to 0.00)
Level change at introduction	**5.29 (2.47 to 8.11)**	-0.04 (-0.10 to 0.01)	**-0.03 (-0.04 to -0.02)**	**-0.03 (-0.05 to -0.02)**	0.005 (-0.02 to 0.03)
Trend change at introduction	**-0.50 (-0.70 to -0.30)**	**0.005 (0.001 to 0.009)**	**0.003 (0.002 to 0.004)**	**0.003 (0.002 to 0.003)**	-0.001 (-0.002 to 0.001)
Level change at withdrawal	1.37 (-1.63 to 4.37)	-0.03 (-0.08 to 0.02)	0.001 (-0.01 to 0.02)	-0.01 (-0.02 to 0.004)	0.01 (-0.01 to 0.03)
Trend change at withdrawal	**0.35 (0.13 to 0.57)**	-0.003 (-0.008 to 0.002)	**-0.002 (-0.003 to -0.001)**	**-0.002 (-0.003 to -0.001)**	0.00 (-0.001 to 0.002)

[1]Values are given to two decimal places or, for values <0.1, one significant figure. Values <0.001 are shown as 0.00; [2]Bold indicates where 95% confidence intervals do not cross 0.

For peer review only

378 **Table 3. Predicted effects (95% CI) of introduction and withdrawal of the Health in Pregnancy Grant at 24 months after each event**

Outcome	21 months after introduction		24 months after withdrawal	
	Absolute change	Relative change (%)	Absolute change	Relative change (%)
Mean gestational age at booking (days)	**-4.8 (-8.2 to -2.3)**[1]	**-6.2 (-10.5 to -3.0)**	**14.0 (2.8 to 16.8)**	**25.2 (2.1 to 33.2)**
Proportion booking by 10 weeks	0.06 (-0.02 to 12.5)	10.3 (-4.2 to 22.6)	-0.14 (-0.24 to 0.03)	-17.4 (-26.8 to 1.3)
Proportion booking by 18 weeks	**0.02 (0.01 to 0.04)**	**2.2 (1.2 to 3.9)**	**-0.08 (-0.08 to -0.02)**	**-7.6 (-7.9 to -2.2)**
Proportion booking by 25 weeks	**0.02 (0.01 to 0.03)**	**1.9 (0.6 to 3.5)**	**-0.09 (-0.09 to -0.03)**	**-8.3 (-8.6 to -3.1)**
Proportion of babies small for gestational age	-0.01 (-0.03 to 0.01)	-8.1 (-25.9 to 10.8)	0.03 (-0.03 to 0.08)	29.8 (-57.4 to 104.9)

379 [1]Bold indicates where 95% confidence intervals do not cross 0

For peer review only

For peer review only - http://bmjopen.bmj.com/site/about/guidelines.xhtml

Table 4. Summary of interactions between parity and the effect of the introduction and withdrawal of the Health in Pregnancy Grant, model coefficients (95% CI),

Model variable	Mean gestational age booking	Proportion booking by 10 weeks	Proportion booking by 18 weeks	Proportion booking by 25 weeks	Proportion of babies SGA
Time	0.03 (-0.01 to 0.08)	-0.00 (-0.00 to 0.00)[1]	-0.00 (-0.00 to 0.00)	-0.00 (-0.00 to 0.00)	-0.00 (-0.00 to 0.00)
Parity >0 vs 0	0.09 (-2.54 to 2.72)	**-0.04 (-0.09 to -0.003)**[2]	0.008 (-0.006 to 0.02)	0.01 (-0.001 to 0.02)	-0.004 (-0.02 to 0.02)
Parity * time	-0.02 (-0.08 to 0.04)	0.00 (-0.00 to 0.001)	0.00 (-0.00 to 0.00)	0.00 (-0.00 to 0.00)	-0.00 (-0.00 to 0.00)
Level change at introduction	**5.57 (1.62 to 9.51)**	0.01 (-0.05 to 0.07)	**-0.04 (-0.06 to -0.01)**	**-0.04 (-0.05 to -0.02)**	0.01 (-0.02 to 0.04)
Trend change at introduction	**-0.50 (-0.78 to -0.21)**	0.003 (-0.001 to 0.008)	**0.002 (0.001 to 0.004)**	**0.003 (0.001 to 0.004)**	-0.001 (-0.004 to 0.001)
Parity * level change at introduction	-0.61 (-6.19 to 4.97)	-0.04 (-0.13 to 0.04)	0.007 (-0.02 to 0.04)	0.007 (-0.02 to 0.03)	-0.01 (-0.06 to 0.03)
Parity * trend change at introduction	0.01 (-0.39 to 0.41)	0.00 (-0.006 to 0.007)	-0.00 (-0.002 to 0.002)	-0.00 (-0.002 to 0.002)	0.002 (-0.002 to 0.005)
Level change at withdrawal	1.03 (-3.16 to 5.22)	-0.02 (-0.08 to 0.05)	0.005 (-0.02 to 0.03)	-0.01 (-0.03 to 0.001)	0.02 (-0.04 to 0.02)
Trend change at withdrawal	0.30 (-0.01 to 0.61)	-0.002 (-0.006 to 0.003)	**-0.002 (-0.003 to -0.00)**	-0.002 (-0.003 to 0.001)	0.001 (-0.002 to 0.003)
Parity * level change at withdrawal	-0.13 (-5.80 to 6.06)	0.006 (-0.09 to 0.10)	-0.003 (-0.04 to 0.03)	0.001 (-0.03 to 0.03)	-0.03 (-0.07 to 0.02)
Parity * trend change at withdrawal	0.08 (-0.36 to 0.52)	-0.00 (-0.008 to 0.006)	-0.00 (-0.003 to 0.002)	-0.00 (-0.002 to 0.002)	-0.001 (-0.004 to 0.003)

[1]Values are given to two decimal places or, for values <0.1, one significant figure. Values <0.001 are shown as 0.00; [2]Bold indicates where 95% confidence intervals do not cross 0.

For peer review only

For peer review only - http://bmjopen.bmj.com/site/about/guidelines.xhtml

Table 5. Summary of interactions between maternal age and the effect of the introduction and withdrawal of the Health in Pregnancy Grant, model coefficients (95% CI)

	Mean gestational age booking	Proportion booking by 10 weeks	Proportion booking by 18 weeks	Proportion booking by 25 weeks	Proportion of babies SGA
Time	-0.01 (-0.06 to 0.05)	0.00[1] (-0.001 to 0.002)	-0.00 (-0.00 to 0.00)	-0.00 (-0.00 to 0.00)	-0.00 (-0.00 to 0.00)
Age group	**-3.41 (-5.39 to -1.42)**[2]	**0.05 (0.002 to 0.11)**	**0.01 (0.004 to 0.02)**	0.00 (-0.006 to 0.007)	**-0.04 (-0.05 to -0.03)**
Age * time	0.03 (-0.01 to 0.08)	-0.00 (-0.001 to 0.001)	-0.00 (-0.00 to 0.00)	-0.00 (-0.00 to 0.00)	0.00 (-0.00 to 0.001)
Level change at introduction	3.02 (-0.87 to 6.90)	-0.04 (-0.10 to 0.02)	-0.03 (-0.05 to -0.001)	**-0.02 (-0.04 to -0.004)**	-0.002 (-0.04 to 0.03)
Trend change at introduction	-0.25 (-0.53 to 0.04)	0.002 (-0.003 to 0.008)	0.002 (-0.00 to 0.004)	**0.002 (0.00 to 0.003)**	0.00 (-0.002 to 0.003)
Age * level change at introduction	2.15 (-0.86 to 5.16)	-0.01 (-0.06 to 0.04)	-0.003 (-0.02 to 0.02)	-0.005 (-0.02 to 0.001)	0.005 (-0.02 to 0.03)
Age * trend change at introduction	**-0.26 (-0.48 to -0.04)**	0.001 (-0.003 to 0.005)	0.001 (-0.00 to 0.002)	0.001 (-0.00 to 0.002)	-0.001 (-0.003 to 0.001)
Level change at withdrawal	0.57 (-3.45 to 4.59)	-0.04 (-0.10 to 0.02)	0.01 (-0.02 to 0.04)	-0.006 (-0.03 to 0.02)	0.007 (-0.03 to 0.05)
Trend change at withdrawal	0.08 (-0.24 to 0.40)	-0.00 (-0.006 to 0.005)	-0.001 (-0.003 to 0.001)	-0.001 (-0.003 to 0.00)	-0.001 (-0.00 to 0.002)
Age * level change at withdrawal	1.23 (-1.88 to 4.34)	0.01 (-0.04 to 0.06)	-0.01 (-0.04 to 0.01)	-0.008 (-0.02 to 0.008)	0.002 (-0.03 to 0.03)
Age * trend change at withdrawal	**0.27 (0.02 to 0.52)**	-0.002 (-0.007 to 0.002)	-0.00 (-0.002 to 0.001)	-0.001 (-0.002 to 0.00)	0.001 (-0.001 to 0.003)

[1]Values are given to two decimal places or, for values <0.1, one significant figure. Values <0.001 are shown as 0.00; [2]Bold indicates where 95% confidence intervals do not cross 0.

For peer review only

For peer review only - http://bmjopen.bmj.com/site/about/guidelines.xhtml

Table 6. Summary of interactions between Index of Multiple Deprivation group and the effect of the introduction and withdrawal of the Health in Pregnancy Grant, model coefficients (95% CI)

	Mean gestational age booking	Proportion booking by 10 weeks	Proportion booking by 18 weeks	Proportion booking by 25 weeks	Proportion of babies SGA
Time	**0.11 (0.03 to 0.19)**[1]	-0.001 (-0.003 to 0.001)	**-0.001 (-0.001 to -0.001)**	-0.00 (-0.00 to 0.00)[2]	0.00 (-0.00 to 0.00)
Deprivation tertile	**4.53 (2.90 to 6.15)**	**-0.09 (-0.13 to -0.05)**	**-0.02 (-0.03 to -0.001)**	-0.005 (-0.01 to 0.003)	**0.04 (0.03 to 0.05)**
Deprivation * time	**-0.04 (-0.07 to -0.00)**	0.00 (-0.00 to 0.001)	0.00 (-0.00 to 0.00)	0.00 (-0.00 to 0.00)	-0.00 (-0.00 to 0.00)
Level change at introduction	**17.18 (9.55 to 24.81)**	-0.06 (-0.17 to 0.06)	**-0.09 (-0.14 to -0.05)**	**-0.10 (-0.13 to -0.06)**	0.01 (-0.05 to 0.07)
Trend change at introduction	**-1.04 (-1.59 to -0.49)**	0.005 (-0.003 to 0.01)	**0.004 (0.001 to 0.007)**	**0.005 (0.002 to 0.008)**	-0.00 (-0.005 to 0.003)
Deprivation * level change at introduction	**-5.02 (-8.56 to -1.49)**	0.007 (-0.05 to 0.06)	**0.03 (0.004 to 0.05)**	**0.03 (0.01 to 0.04)**	-0.001 (-0.03 to 0.03)
Deprivation * trend change at introduction	0.22 (-0.03 to 0.47)	-0.00 (-0.004 to 0.004)	-0.001 (-0.002 to 0.001)	-0.001 (-0.002 to -0.00)	0.00 (-0.002 to 0.002)
Level change at withdrawal	6.47 (-1.68 to 14.62)	-0.06 (-0.18 to 0.06)	-0.008 (-0.06 to 0.04)	-0.03 (-0.07 to 0.01)	0.02 (-0.04 to 0.08)
Trend change at withdrawal	0.59 (-0.005 to 1.18)	-0.002 (-0.01 to 0.01)	-0.002 (-0.006 to 0.001)	**-0.003 (-0.006 to -0.00)**	-0.001 (-0.006 to 0.003)
Deprivation * level change at withdrawal	-2.10 (-5.88 to 1.67)	0.01 (-0.04 to 0.07)	0.004 (-0.02 to 0.03)	0.008 (-0.01 to 0.03)	-0.003 (-0.03 to 0.03)
Deprivation * trend change at withdrawal	-0.10 (-0.37 to 0.18)	-0.00 (-0.005 to 0.005)	0.00 (-0.002 to 0.002)	0.00 (-0.001 to 0.002)	0.00 (-0.001 to 0.003)

[1]Bold indicates where 95% confidence intervals do not cross 0; [2]values are given to two decimal places or, for values <0.1, one significant figure. Values <0.001 are shown as 0.00.

For peer review only

For peer review only - http://bmjopen.bmj.com/site/about/guidelines.xhtml

Figure 1. Summary of interrupted time series models of the effect of the introduction and withdrawal of the Health in Pregnancy Grant

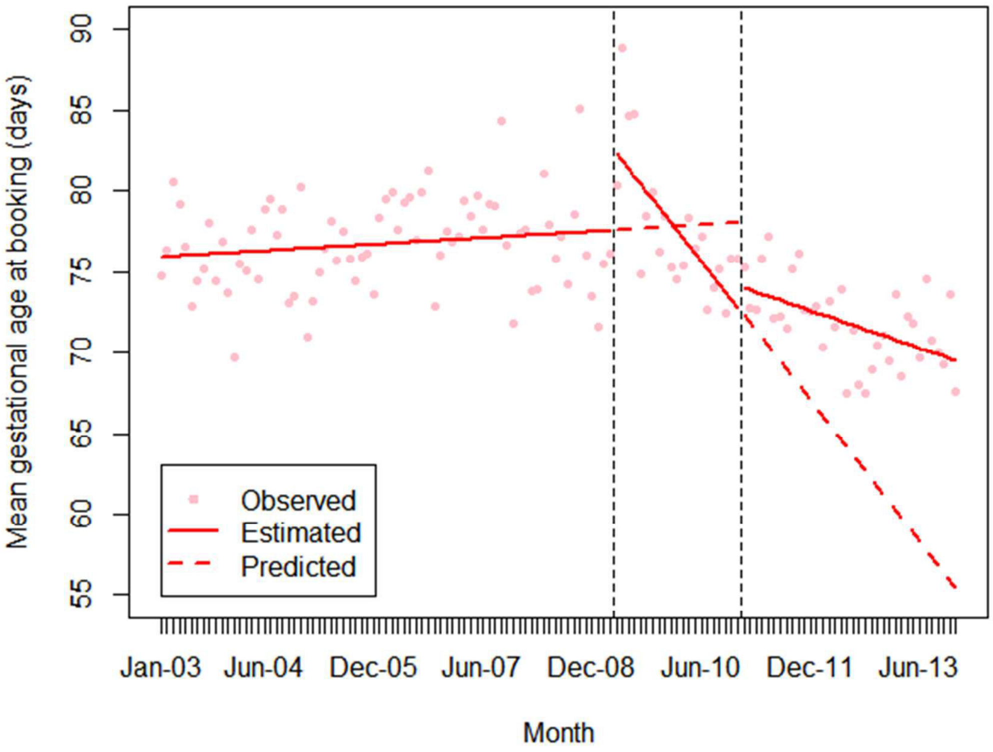

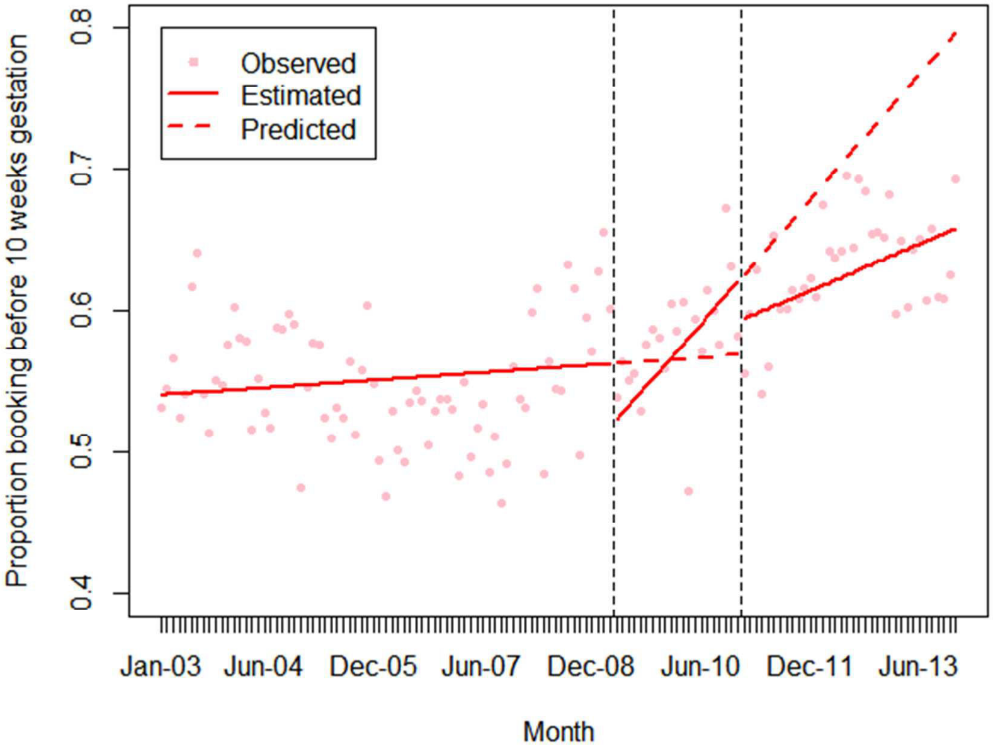

Fig 1. cont.

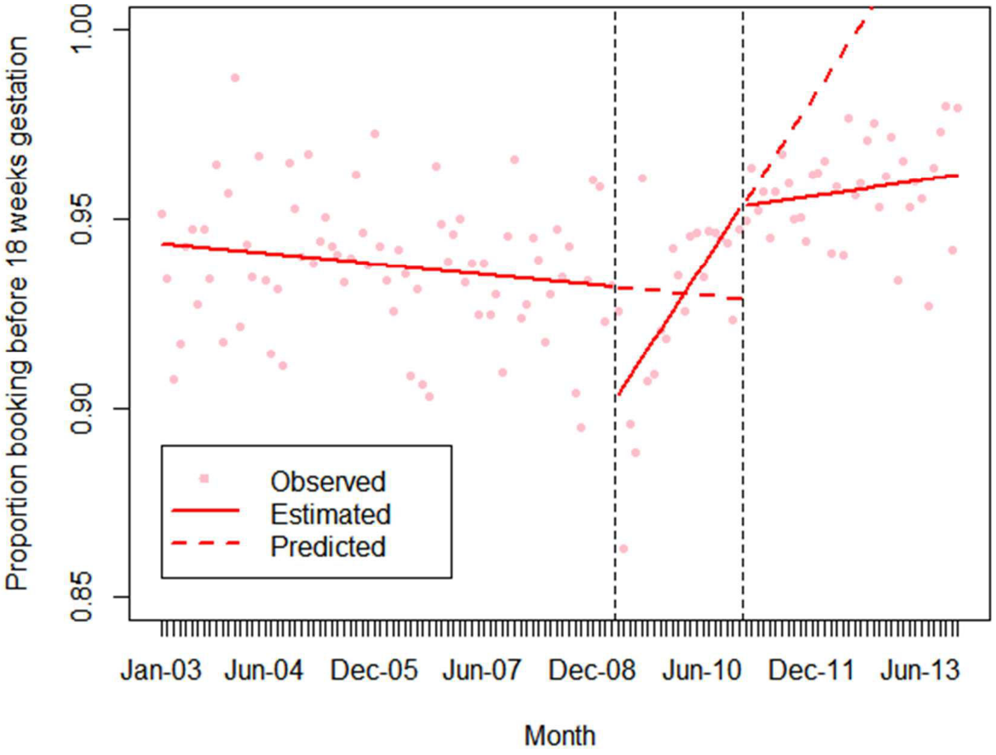
Proportion booking before 18 weeks gestation
1.00
0.95
0.90
0.85
Observed
Estimated
Predicted
Jan-03
Jun-04
Dec-05
Jun-07
Dec-08
Jun-10
Dec-11
Jun-13
Month

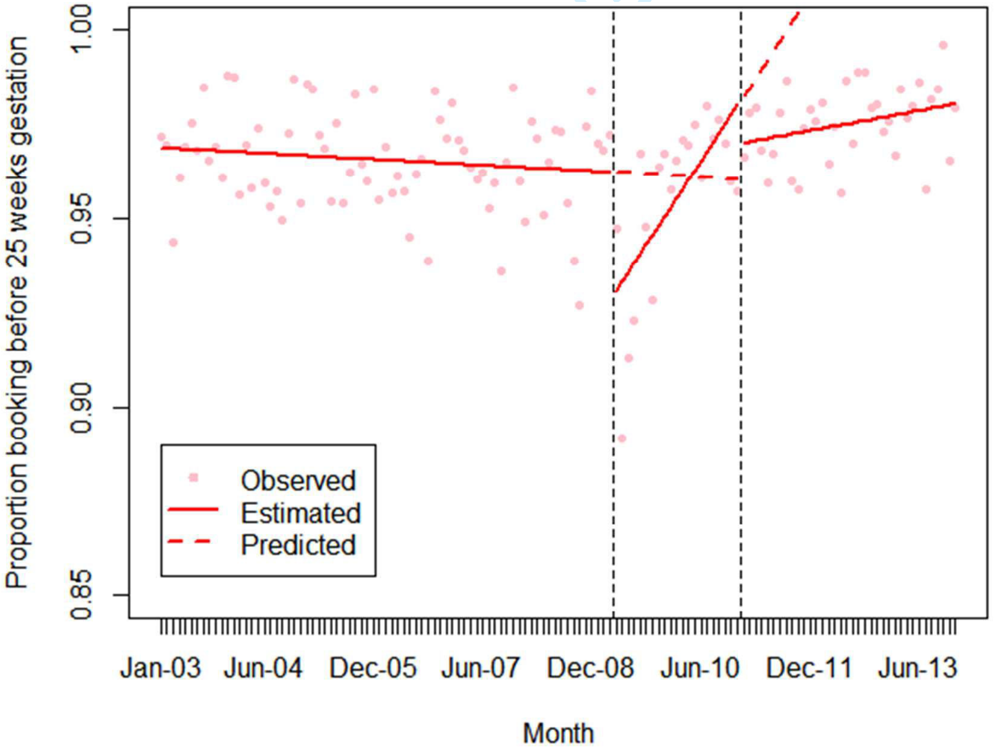
Proportion booking before 25 weeks gestation
1.00
0.95
0.90
0.85
Observed
Estimated
Predicted
Jan-03
Jun-04
Dec-05
Jun-07
Dec-08
Jun-10
Dec-11
Jun-13
Month

399 **Fig 1. cont.**

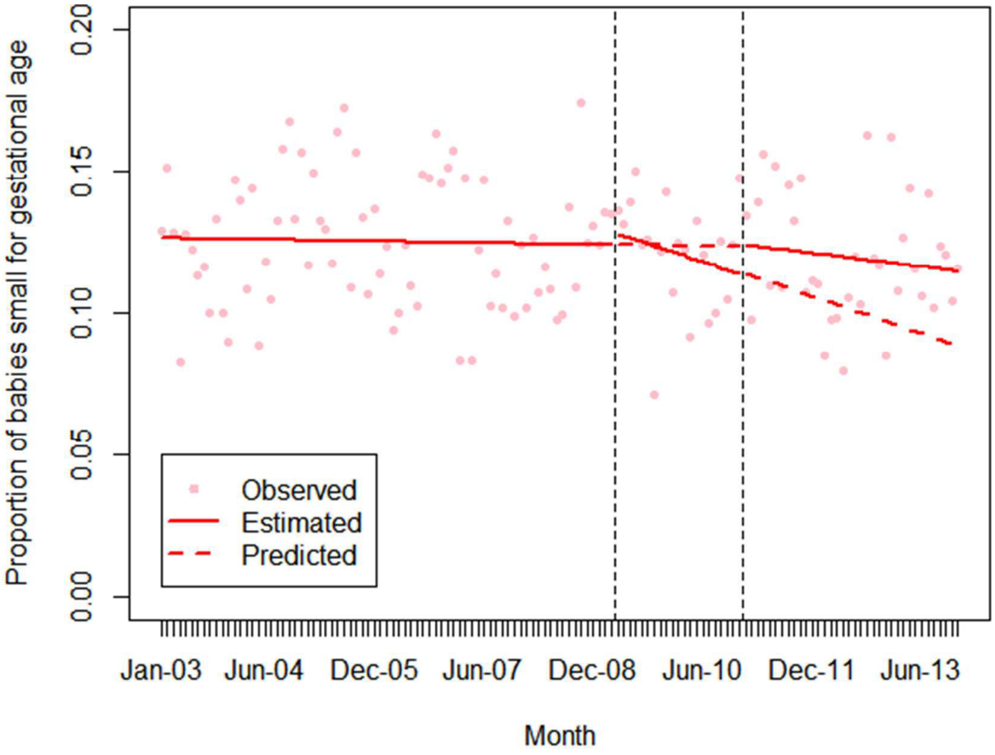

400

review only

Fig 2. Summary of interrupted time series models, interactions between maternal age group and the effect of the introduction and withdrawal of the Health in Pregnancy Grant

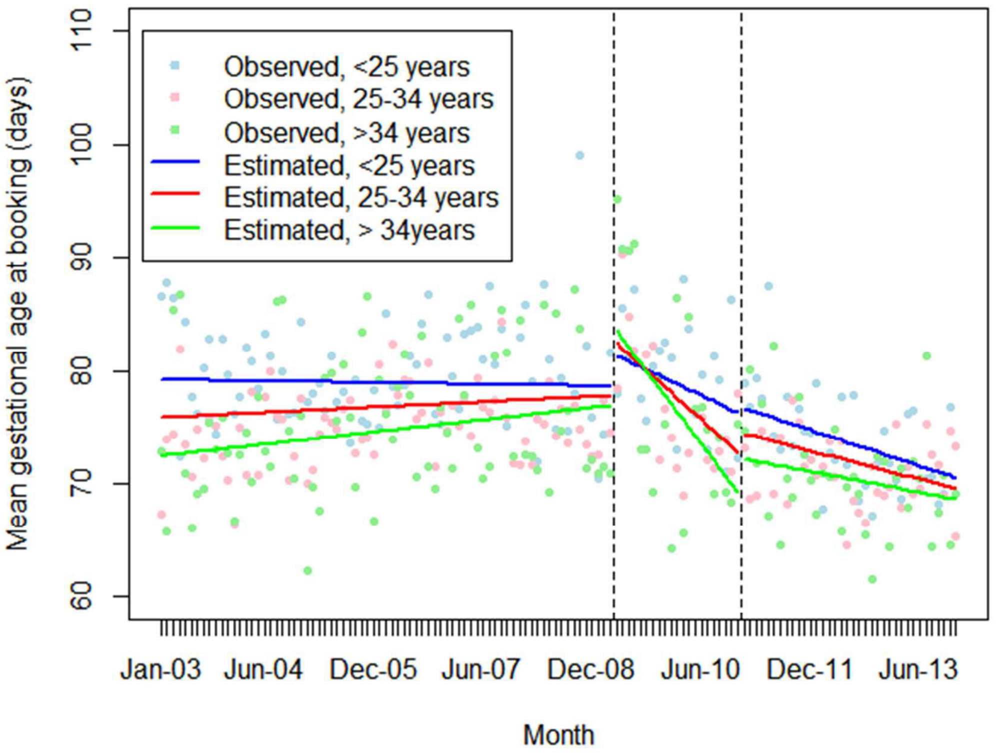

Fig 3. Summary of interrupted time series models, interactions between Index of Multiple Deprivation group and the effect of the introduction and withdrawal of the Health in Pregnancy Grant

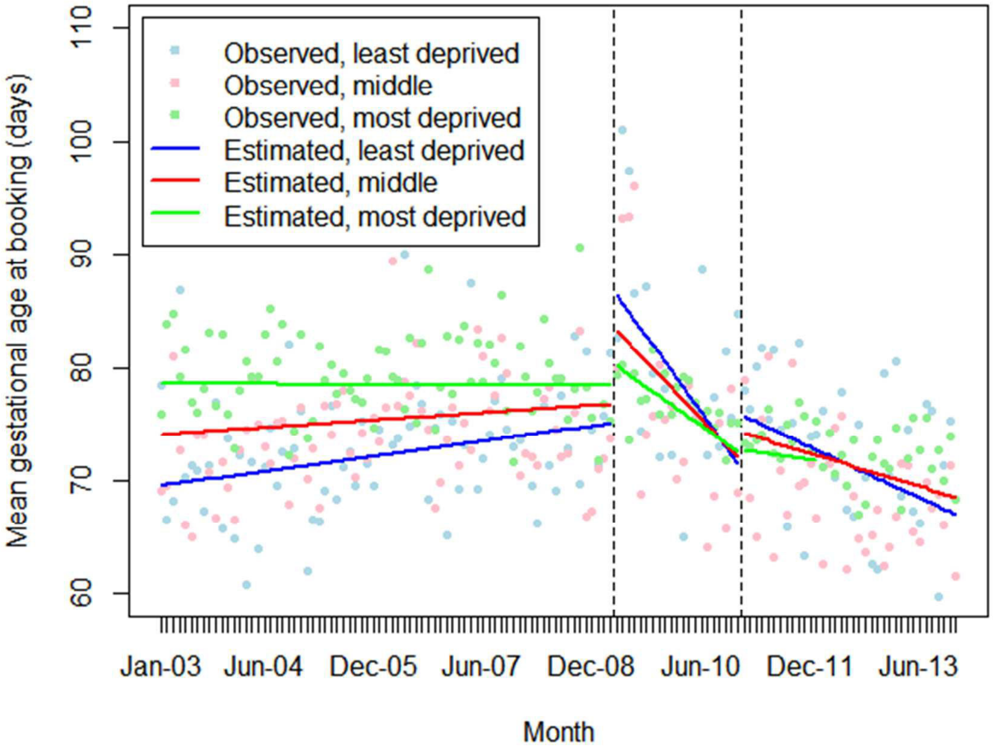

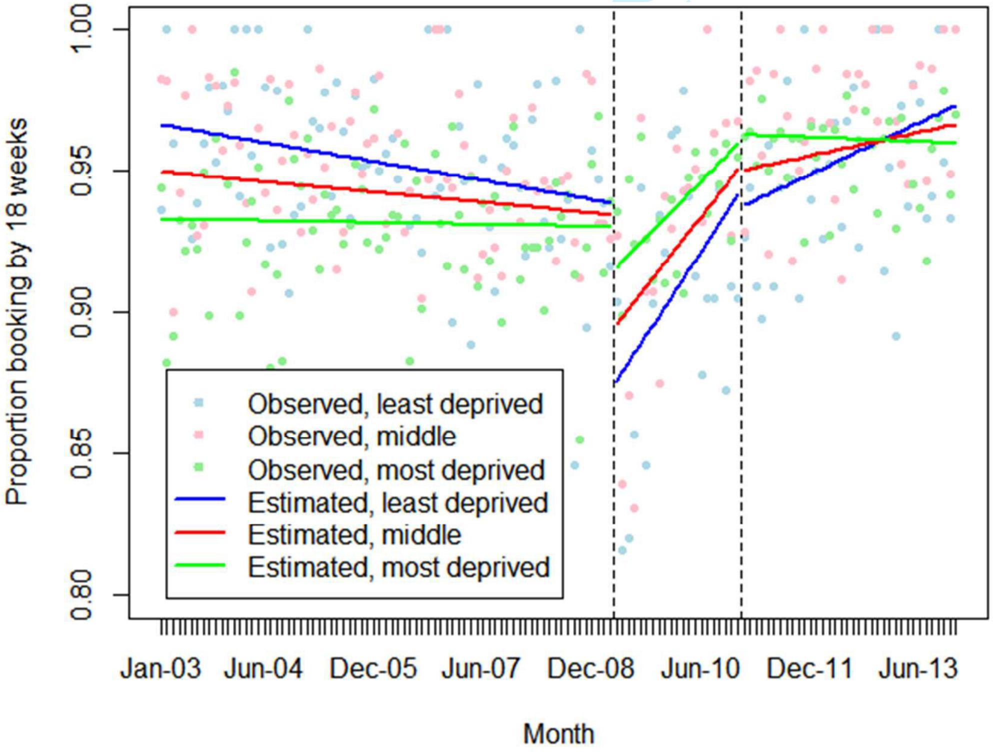

408 **Fig 3. Cont.**

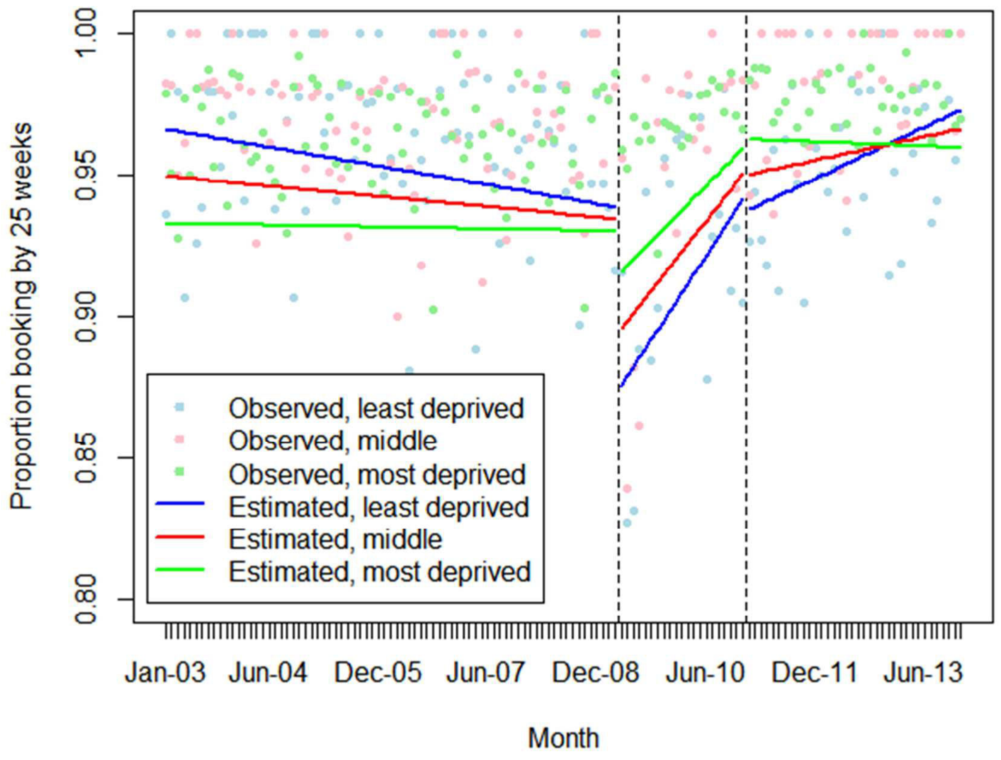

409

For peer review only - http://bmjopen.bmj.com/site/about/guidelines.xhtml

STROBE Statement—checklist of items that should be included in reports of observational studies

	Item No	Recommendation	Page/line ref
**Title and abstract**	1	(*a*) Indicate the study's design with a commonly used term in the title or the abstract	p1, lines 2-3
		(*b*) Provide in the abstract an informative and balanced summary of what was done and what was found	p2, lines 17-37
**Introduction**			
Background/rationale	2	Explain the scientific background and rationale for the investigation being reported	p4-5, lines 58-84
Objectives	3	State specific objectives, including any prespecified hypotheses	p5, lines 85-88
**Methods**			
Study design	4	Present key elements of study design early in the paper	p5, line 90
Setting	5	Describe the setting, locations, and relevant dates, including periods of recruitment, exposure, follow-up, and data collection	p5-6, lines 92-106
Participants	6	(*a*) *Cohort study*—Give the eligibility criteria, and the sources and methods of selection of participants. Describe methods of follow-up<br>*Case-control study*—Give the eligibility criteria, and the sources and methods of case ascertainment and control selection. Give the rationale for the choice of cases and controls<br>*Cross-sectional study*—Give the eligibility criteria, and the sources and methods of selection of participants	p5-6, lines 92-106
		(*b*) *Cohort study*—For matched studies, give matching criteria and number of exposed and unexposed<br>*Case-control study*—For matched studies, give matching criteria and the number of controls per case	No matching
Variables	7	Clearly define all outcomes, exposures, predictors, potential confounders, and effect modifiers. Give diagnostic criteria, if applicable	Exposures – p5, lines 95-102<br>Outcomes – p6, lines 106-114<br>Potential effect modifiers – p6, lines 116-121
Data sources/ measurement	8*	For each variable of interest, give sources of data and details of methods of assessment (measurement). Describe comparability of assessment methods if there is more than one group	p5-6, lines 92-121
Bias	9	Describe any efforts to address potential sources of bias	p6-7, lines 123-127; p10-11, lines 216-220
Study size	10	Explain how the study size was arrived at	p5, lines 95-101
Quantitative variables	11	Explain how quantitative variables were handled in the analyses. If applicable, describe which groupings were chosen and why	p6-7, lines 114-141

For peer review only - http://bmjopen.bmj.com/site/about/guidelines.xhtml

Statistical methods	12	(*a*) Describe all statistical methods, including those used to control for confounding	p7, lines 127-141
		(*b*) Describe any methods used to examine subgroups and interactions	p7, lines 139-140
		(*c*) Explain how missing data were addressed	p6-7, lines 121-125
		(*d*) *Cohort study*—If applicable, explain how loss to follow-up was addressed<br>*Case-control study*—If applicable, explain how matching of cases and controls was addressed<br>*Cross-sectional study*—If applicable, describe analytical methods taking account of sampling strategy	Not applicable
		(*e*) Describe any sensitivity analyses	None – see p10-11, lines 214-218
**Results**			
Participants	13*	(a) Report numbers of individuals at each stage of study—eg numbers potentially eligible, examined for eligibility, confirmed eligible, included in the study, completing follow-up, and analysed	Table 1
		(b) Give reasons for non-participation at each stage	Table 1
		(c) Consider use of a flow diagram	Not considered helpful
Descriptive data	14*	(a) Give characteristics of study participants (eg demographic, clinical, social) and information on exposures and potential confounders	Table 1
		(b) Indicate number of participants with missing data for each variable of interest	Table 1
		(c) *Cohort study*—Summarise follow-up time (eg, average and total amount)	Not applicable
Outcome data	15*	*Cohort study*—Report numbers of outcome events or summary measures over time	Not applicable
		Case-control study—Report numbers in each exposure category, or summary measures of exposure	Not applicable
		Cross-sectional study—Report numbers of outcome events or summary measures	Table 1
Main results	16	(*a*) Give unadjusted estimates and, if applicable, confounder-adjusted estimates and their precision (eg, 95% confidence interval). Make clear which confounders were adjusted for and why they were included	Tables 2-6
		(*b*) Report category boundaries when continuous variables were categorized	Table 1
		(*c*) If relevant, consider translating estimates of relative risk into absolute risk for a meaningful time period	Table 3
Other analyses	17	Report other analyses done—eg analyses of subgroups and interactions, and sensitivity analyses	Tables 4-6
**Discussion**			
Key results	18	Summarise key results with reference to study objectives	p9-10, lines 183-195
Limitations	19	Discuss limitations of the study, taking into account sources of potential bias or imprecision. Discuss both direction and magnitude of any potential bias	p10, lines 197-212
Interpretation	20	Give a cautious overall interpretation of results considering objectives, limitations, multiplicity of analyses, results from similar studies, and other relevant evidence	p10-12, lines 214-244
Generalisability	21	Discuss the generalisability (external validity) of the study results	p10, lines 211-212

For peer review only - http://bmjopen.bmj.com/site/about/guidelines.xhtml

**Other information**			
Funding	22	Give the source of funding and the role of the funders for the present study and, if applicable, for the original study on which the present article is based	p13-14, lines 276-288

*Give information separately for cases and controls in case-control studies and, if applicable, for exposed and unexposed groups in cohort and cross-sectional studies.

Note: An Explanation and Elaboration article discusses each checklist item and gives methodological background and published examples of transparent reporting. The STROBE checklist is best used in conjunction with this article (freely available on the Web sites of PLoS Medicine at http://www.plosmedicine.org/, Annals of Internal Medicine at http://www.annals.org/, and Epidemiology at http://www.epidem.com/). Information on the STROBE Initiative is available at www.strobe-statement.org.

For peer review only

For peer review only - http://bmjopen.bmj.com/site/about/guidelines.xhtml

BMJ Open

Associations between introduction and withdrawal of a financial incentive and timing of attendance for antenatal care and incidence of small for gestational age: natural experimental evaluation using interrupted time series methods

Journal:	*BMJ Open*
Manuscript ID	bmjopen-2017-017697.R1
Article Type:	Research
Date Submitted by the Author:	21-Nov-2017
Complete List of Authors:	Adams, J; University of Cambridge, Centre for Diet and Activity Research, MRC Epidemiology Unit<br>van der Waal, Zelda; Newcastle University<br>Rushton, Steven; Newcastle University<br>Rankin, Judith; Newcastle University, Institute of Health & Society
**Primary Subject Heading**:	Public health
Secondary Subject Heading:	Obstetrics and gynaecology, Health policy
Keywords:	Health policy < HEALTH SERVICES ADMINISTRATION & MANAGEMENT, PUBLIC HEALTH, OBSTETRICS, PREVENTIVE MEDICINE, Community child health < PAEDIATRICS

SCHOLARONE™
Manuscripts

For peer review only

Associations between introduction and withdrawal of a financial incentive and timing of attendance for antenatal care and incidence of small for gestational age: natural experimental evaluation using interrupted time series methods

Jean Adams, PhD;[1*] Zelda van der Waal, PhD;[2] Steve Rushton, PhD;[2] Judith Rankin, PhD[3]

[1]MRC Epidemiology Unit, University of Cambridge School of Clinical Medicine, Box 285 Institute of Metabolic Science, Cambridge Biomedical Campus, Cambridge CB2 0QQ, UK

[2]School of Biology, Ridley Building 2, Newcastle University, Claremont Road, Newcastle upon Tyne, NE1 7RU, UK

[3]Institute of Health & Society, Baddiley Clark Building, Newcastle University, Richardson Road, Newcastle upon Tyne, NE2 4AX, UK

*author for correspondence: jma79@medschl.cam.ac.uk; tel +44 1223 769 142

Keywords: reward, incentive, maternal, antenatal, prenatal, natural experiment

For peer review only

Abstract

Objectives

To determine whether introduction or withdrawal of a maternal financial incentive was associated with changes in timing of first attendance for antenatal care ('booking'), or incidence of small for gestational age.

Design

A natural experimental evaluation using interrupted time series analysis.

Setting

A hospital-based maternity unit.

Participants

34,589 women (and their live-born babies) who delivered at the study hospital and completed the 25th week of pregnancy in the 75 months before (January 2003–March 2009), 21 months during (April 2009–December 2010), and 36 months after (January 2011–December 2013) the incentive was available.

Intervention

The Health in Pregnancy Grant was a financial incentive of £190 ($235; €211) payable to pregnant women in the UK from the 25th week of pregnancy, contingent on them receiving routine antenatal care.

Primary and secondary outcome measures

The primary outcome was mean gestational age at booking. Secondary outcomes were proportion of women booking by 10, 18 and 25 weeks gestation; and proportion of babies that were small for gestational age.

For peer review only

For peer review only - http://bmjopen.bmj.com/site/about/guidelines.xhtml

Results

By 21 months after introduction of the grant (i.e. immediately prior to withdrawal), compared to what was predicted given prior trends, there was an reduction in mean gestational age at booking of 4·8 days (95% confidence intervals: 2·3 to 8·2). The comparable figure for 24 months after withdrawal was an increase of 14·0 days (95%CI: 2·8 to 16·8). No changes in incidence of small for gestational age babies were seen.

Conclusions

The introduction of a universal financial incentive for timely attendance at antenatal care was associated with a reduction in mean gestational age at first attendance, but not proportion of babies that were small for gestational age. Future research should explore the effects of incentives offered at different times and of differing values; and how stakeholders view such incentives.

For peer review only

Article summary

Strengths and limitations of this study

- We used interrupted time series methods to evaluate this natural experiment; one of the strongest quasi-experimental research designs available.
- By including substantial data before and after interventions, we took account of underlying secular trends.
- However, interrupted time series designs are observational and we cannot categorically ascribe the changes documented to the intervention.
- One of our secondary outcomes was proportion of babies born small for gestational age - a substantial improvement on previous studies that use a simple low birth weight cut-off.
- Differences between women included and excluded from the analyses may limit external validity, as may our use of data from only one hospital.

For peer review only

Introduction

Financial incentives are increasingly used to encourage health promoting behaviours. However, few large, pragmatic, evaluations in high-income countries have been conducted.[1 2]

The Health in Pregnancy Grant (HiPG) was introduced in April 2009 as a one-off payment of £190 ($235; €211) payable to all pregnant women, normally resident in the UK, after the 25th week of pregnancy, but before delivery. Women submitted a claim form, signed by their doctor or midwife confirming their expected delivery date and that they had received usual antenatal care.[3] A key aim of the HiPG was to act as an "incentive to seek the recommended health advice at the appropriate time".[3] Following a general election in 2010, the HiPG was withdrawn with women only able to claim if they reached the end of the 25th week of pregnancy before 1 January 2011.

England compares poorly to other European countries on perinatal outcomes.[4] One possible reason is poor attendance at antenatal care, which is associated with increased risk of small for gestational age (SGA),[5 6] and a range of adverse outcomes.[7-9] National guidance recommends that the first antenatal (or 'booking') visit should ideally take place by 10 weeks gestation and, at the latest, by 18 weeks.[10] Women living in more deprived circumstances tend to book later in pregnancy'.[11]

Health promoting financial incentives may be more effective in promoting one-off behaviours than complex behaviour change.[12-15] Antenatal care is a series of one-off behaviours and may be particularly responsive to incentives. However, a recent systematic review found only five trials of maternal incentives for antenatal care – three conducted in the USA and one each in Mexico and Honduras.[16] No effect on timing of antenatal care was found (although only one study investigated this).[17] No studies included birthweight or SGA as outcomes. A further observational study from the USA found no effect of an incentive on incidence of low birth weight.[18] One recent evaluation of the HiPG in Scotland reported no effect on birth weight, but a positive effect on the proportion of women booking by 25 weeks (other aspects of timing of attendance were not studied).[19]

For peer review only - http://bmjopen.bmj.com/site/about/guidelines.xhtml

UK public health policy makers and members of the public think that it may be appropriate to target financial incentives at people living in more deprived circumstances – perhaps because those living in more deprived circumstances are more in need of financial support.[20 21] There is some systematic review evidence that people living in more deprived circumstances may be more responsive to fiscal interventions in general. Other personal characteristics, such as age and previous experience of the behaviour incentivised, may also influence responsiveness. However, differential responses to health promoting financial incentives between population groups have not been systematically studied.[1]

The introduction and withdrawal of the HiPG provided a unique opportunity for a large-scale, pragmatic, natural experimental evaluation of a health promoting financial incentive.[23] Our research questions were: was the introduction or withdrawal of the HiPG associated with a change in the timing of booking, or incidence of SGA? Did any effect of the HiPG vary according to maternal age, parity or deprivation?

Methods

We used an interrupted time series (ITS) design.

Data and inclusion criteria

We used routine data from a maternity unit in a tertiary hospital in northern England, extracted in May 2015. The study hospital is a general teaching hospital with over 1000 beds in a town with a population of ~175,000 people. Both the town and surrounding areas are more deprived than the English average.

Participants were women (and their live-born babies) who delivered at the study hospital and were known to have completed the 25th week of pregnancy in the 75 months before (January 2003–March 2009) introduction of the HiPG, 21 months during (April 2009–December 2010) availability of the HiPG, and 36 months after (January 2011–December 2013) withdrawal of the HiPG. The time periods included were pragmatically arrived at based on when data was available from, and when the HiPG was introduced and withdrawn. Our final data set of 132 monthly data points (and a mean of 262 cases per data point – see results section), substantially exceeds the minimum requirements for ITS (of at least 8 data points per

For peer review only

intervention phase and 100 individual observations per data point).[24] Aggregating to the weekly, rather than monthly, level would not have achieved these requirements – with a mean of 60 cases in each of 572 weekly data points. As calculation of when women reached the 25th week of pregnancy depended on knowing the date of their last menstrual period (LMP), women for whom this date was missing were excluded.

Women who had a termination or experienced a stillbirth were excluded, as were women with missing data on any variable of interest. Women who delivered more than one live baby in any one pregnancy, or had more than one pregnancy that resulted in a live birth during the study period, were included with each baby counted as a separate 'case'. As we did not have access to any identifiable data on women, we were not able to determine on how many occasions this occurred or to take it into account in modelling.

Outcome measures

Our outcome measures focus on the stated aim of the HiPG – to encourage women to "seek the recommended health advice at the appropriate time".[3] The primary outcome was mean gestational age at booking, calculated from dates of booking (recorded by antenatal care staff) and LMP (self-reported). As national guidance recommends booking ideally before 10 weeks, and definitely before 18 weeks, and the HiPG was available to women from the 25th week, the proportion of women booking by 10, 18 and 25 weeks gestation were secondary outcomes.[10]

As timely attendance for antenatal care is thought to improve perinatal outcomes, we included a final secondary outcome: proportion of babies that were SGA. It should be noted that there is likely to be a long and complicated chain of causation, if any, between receiving the HiPG and changes in gestational weight for age. We defined SGA as birth weight z-score below the 10th percentile for sex-specific gestational age.[25] This was calculated using infant sex, birth weight and dates of LMP and delivery (all except LMP recorded by antenatal care and delivery staff).

For peer review only

124 *Other variables of interest*

125 We studied whether any effects of the HiPG on the outcomes varied according to maternal age at delivery (in years, calculated from maternal date of birth and date of delivery and divided into three groups: <25, 25-34, or 35+ years), parity (self-reported and considered as 0 or 1+ in analyses) and socio-economic position. The main age group (age 25-34 years) was coded using mid-decade to mid-decade as the convention recommended to increase comparability between studies. We did not further sub-divide the other age groups as only 8 women in the included sample were aged less than 15 years and only 27 were aged more than 44 years. Socio-economic position was measured using the Index of Multiple Deprivation (IMD) 2007 rank assigned to maternal address at delivery.[26] IMD is an area-based measure of deprivation and ranks were divided into thirds for analysis based on the distribution across England.

134 *Data preparation*

135 Data cleaning aimed to exclude data that were implausible. Date of LMP was recorded as month and year only in around 20% of cases. To include these cases, day of month was set to the 1st. Gestational age at first antenatal care of less than 28 days (4 weeks) or more than 308 days (44 weeks), gestational age at delivery of less than 24 weeks or more than 44 weeks, or birth weight z-scores of less than -3 or more than 3 were recoded as missing as these are likely to represent recording or transcriptions errors.[25]

140 *Data analyses*

141 We first compared women in the dataset who did and did not meet the inclusion criteria using χ^2 and t-tests.

142 For the main analysis, an uncontrolled, multiple time points, ITS design was used. The unit of analysis was the month in which women entered the 25th week of pregnancy. ITS models estimate the change in 'level' and 'trend' of the outcome of interest associated with the intervention. The change in level is the difference in intercepts between regression lines estimated from observations before and after the intervention. The change in trend is the difference in slopes. In the case of two 'interventions' (e.g. introduction and withdrawal of the HiPG), two changes in level and trend are estimated.

For peer review only - http://bmjopen.bmj.com/site/about/guidelines.xhtml

Generalised least squares models were used allowing for autoregressive and moving average correlation structures as appropriate. These allow any effect of periodicity to be taken into account. Firstly, associations between introduction and withdrawal of the HiPG and the outcomes of interest were assessed in the whole cohort, using separate models for each outcome. Final models were used to calculate estimated absolute and relative effects on each outcome of the introduction of the HiPG at 21 months post-implementation (immediately prior to withdrawal), and 24 months after withdrawal, with 95% confidence intervals.[27] Interaction terms were then used to determine whether the effects of the introduction or withdrawal of the HiPG varied by maternal age group, parity or IMD tertile.

Data preparation was conducted in StataSE v14; data analysis in R v3.3.1 and RStudio v0.99.903. We used 95% confidence intervals and a p-value of <0.05 to indicate statistical significance throughout.

Results

Sample description

Of 39,571 women who delivered at the study hospital and were known to have reached the 25th week of gestation between 1st January 2003 and 31st December 2013, full data were available for 34,589 (87.4%). Characteristics of those for did and did not meet the inclusion criteria and hence were included or excluded from the analysis are described in Table 1. Most exclusions were due to missing information on birth weight. Typically, women included in the analyses were aged 25-34 years, of parity 1 or more, lived in the most deprived third of areas in England, and booked by 10 weeks gestation. Women excluded from the analyses tended to be younger, live in more deprived areas, and booked later in their pregnancies than women included. Similar differences between women included and excluded from the analyses were seen in each of the three study periods.

Sample-wide changes in outcomes associated with introduction and withdrawal of the HiPG

Final models for each outcome are summarised in Table 2 and plotted in Figure 1, Figure 2, Figure 3, Figure 4 and Figure 5. Introduction of the HiPG was associated with an immediate increase in mean gestational age at

For peer review only - http://bmjopen.bmj.com/site/about/guidelines.xhtml

172 booking, and a decrease in the proportion booking by 18 and 25 weeks. That is, the immediate effect was for
173 these outcomes to get clinically 'worse'. However, introduction of the HiPG was also associated with an
174 improvement in the trend in mean gestational age at booking and proportion booking by 10, 18 and 25
175 weeks. That is, the longer term effect was a change in trend of these outcomes towards greater clinical
176 improvement over time.

177 Withdrawal of the HiPG was not associated with any level changes in outcomes. However, it was associated
178 with a change in trend in mean gestational age at booking and proportion booking by 18 and 25 weeks
179 towards less clinical improvement over time. The introduction or withdrawal of the HiPG was not associated
180 with any changes in the level or trend in the proportion of babies who were SGA.

181 Table 3 shows the absolute and relative impact of the introduction and withdrawal of the HiPG on each
182 outcome at 21 months post introduction and 24 months post withdrawal. By 21 months after introduction of
183 the HiPG, compared to the counterfactual of what was predicted given trends prior to the introduction of
184 the HiPG, there was a reduction in mean gestational age at booking of 4.8 days (95% confidence intervals:
185 2.3 to 8.2), an increase in the proportion of women booking by 18 weeks of 2.2% (95%CI: 1.2 to 3.9), and an
186 increase in the proportion of women booking by 25 weeks of 1.9% (95%CI: 0.6 to 3.5). Compared to the
187 counterfactual of what was predicted to occur given trends when the HiPG was available, by 24 months after
188 withdrawal, there was an increase in mean gestational age at booking of 14.0 days (95% CI: 2.8 to 16.8), a
189 decrease in the proportion of women booking by 18 weeks of 7.6% (95% CI: 2.2 to 7.9), and a decrease in the
190 proportion of women booking by 25 weeks of 8.3% (95% CI: 3.1 to 8.6).

191 *Differential changes in outcomes associated with introduction and withdrawal of the HiPG across population*
192 *sub-groups*

193 Models including interaction terms for maternal age, parity and IMD tertile are summarised in Tables 4-6.
194 There were no interactions with parity. The associations between introduction and withdrawal of the HiPG
195 and trend in mean gestational age at booking varied by age group (Figure 6), with greater changes in trend in
196 older women.

For peer review only - http://bmjopen.bmj.com/site/about/guidelines.xhtml

197 The association between introduction of the HiPG and mean gestational age at booking and proportion booking by 18 and 25 weeks varied by IMD group (Figure 7, Figure 8 and Figure 9). The introduction of the HiPG was associated with a progressively larger level change (towards older gestational age at booking, and lower proportion booking by 18 or 25 weeks) as deprivation decreased.

201 **Discussion**

202 *Statement of principal findings*

203 This is the first evaluation of the HiPG in England, the first evaluation of a financial incentive for attendance at antenatal care on incidence of SGA, and one of the largest pragmatic evaluations of a health promoting financial incentive in a high-income country. Introduction of the HiPG was associated with immediate deteriorations in timing of booking, but longer term improvements over time. By 21 months after introduction of the HiPG (immediately prior to its withdrawal), mean gestational age at booking had decreased by 4.8 days compared to what would have been expected had it not been introduced. Withdrawal of the HiPG was not associated with any immediate changes in timing of booking, but it was associated with longer-term deteriorations in timing of booking over time. By 24 months after withdrawal, mean gestational age at booking had increased by 14.0 days compared to what would have been expected had it not been withdrawn. We found no association between introduction or withdrawal of the HiPG and incidence of SGA. Trends in outcomes associated with the HiPG did not vary by parity. The positive association between introduction of the HiPG and gestational age at booking was greater in older women. The negative association between introduction of the HiPG and timing of booking was more pronounced in less deprived groups.

217 *Strengths and weaknesses of methods*

218 The ITS approach is one of the strongest quasi-experimental research designs.[24 28] By including substantial data before and after interventions, we took account of underlying secular trends. By studying outcomes at the population-, rather than individual-, level confounding by individual-level variables was avoided. Our

large data set with 132 monthly data points, substantially exceeds the minimum requirements for ITS.[28] By including auto-regressive and moving-average functions, any biases introduced by the serial nature of the data (including seasonality and other periodicities) were accounted for. However, ITS designs are observational and we cannot categorically ascribe the changes documented to the HiPG. Whilst we are not aware of any co-interventions likely to have influenced the outcomes concurrent with the HiPG, it is difficult to absolutely exclude these.

A major strength of our study is the use of SGA. Unlike a simple cut-off for low birth weight, SGA allows sex and gestational age differences in birth weight to be taken into account.

The data we used is likely to contain recording, reporting and transcription errors. Some of these may have introduced bias. 'Feasibility' limits were used for some variables and may have led to misclassification.

Cases included in the analytical cohort differed from those excluded. However, as differences between women included and excluded from the analyses were similar in all three study periods, this is unlikely to introduce bias and so we did impute missing data. Differences between women included and excluded from the analyses may limit external validity, as may our use of data from only one hospital.

Interpretation of findings

Our finding that the introduction of the HiPG was associated with an immediate deterioration in timing of booking may reflect an implementation phase, where the process for obtaining the HiPG was not yet fully understood. For instance, women may have thought that they were only entitled to the HiPG if they delayed attending until after the 25th week of pregnancy. In fact this was not the case – although women could not claim the grant until after the 25th week of pregnancy, whether they had first attended before this had no impact on their entitlement. Whilst we could have conducted further analyses excluding an implementation period (e.g. four months after introduction of the HiPG), this would have been *post-hoc* justified.

The longer-term associations of the introduction of the HiPG on markers of timing at booking are in line with the intention of the intervention – that women should attend for antenatal care earlier in their pregnancies.

For peer review only - http://bmjopen.bmj.com/site/about/guidelines.xhtml

One previous study found no effect on timing of first attendance of providing a voucher for a taxi journey to the antenatal clinic,[17] whilst a further evaluation of the HiPG found it was associated with a positive effect on the proportion of women booking by 25 weeks that disappeared after withdrawal.[19] The substantial difference in incentive value of the HiPG, compared to previous incentives, may explain these differences.

The finding that withdrawal of the HiPG was associated with deterioration of the benefits of its introduction on timing of booking is also not unexpected. On the whole, different women would have been pregnant when the HiPG was and was not available, meaning sustained effects would be highly unlikely.

Changes in timing of attendance for antenatal care associated with the introduction and withdrawal of the HiPG did not translate into differences in the proportion of SGA babies. This may be because the effect size (of 4.8 days at 21 months) was too small to impact on SGA. Two previous studies that examined the effect of incentives for antenatal care on incidence of low birth weight (rather than SGA) also reported no effect.[18 19]

Although the HiPG was only available from the 25th week of pregnancy, we found that its introduction was associated with changes in the proportion of women booking by both 10 and 18 weeks. This indicates that the impact of health promoting financial incentives may not be as specific as previously thought.[29] The HiPG may have been associated with a larger effect on timing of booking if it had been contingent on booking earlier in pregnancy.

We did not find any evidence that the associations of the introduction or withdrawal of the HiPG with the outcomes studied varied by parity. This suggests that prior experience of antenatal care did not diminish the impact of the HiPG. However, the association of the introduction of the HiPG with improvements in gestational age at booking over time were greater in older women. This suggests that age may be a determinant of responsiveness to financial incentives in this context, with older women being more responsive to the intervention. Our data are consistent with the suggestion that those with fewer resources are particularly responsive to incentives.[30] Whilst introduction of the HiPG was associated with an immediate negative change in timing of first antenatal care, this was least pronounced in women living in the most deprived areas.

For peer review only - http://bmjopen.bmj.com/site/about/guidelines.xhtml

Implications of findings for policy, practice and research

It is possible that larger incentives, contingent on attendance earlier than 25 weeks, may have greater impacts on timing of antenatal care and clinical outcomes than seen here. Future research could explore how effects on antenatal care attendance vary with incentive value and timing.

As we used routine data in a retrospective analysis conducted more than two years after withdrawal of the HiPG, we were unable to explore how women and other stakeholders responded to the HiPG. In particular, we do not know what women spent the HiPG on, how doctors and midwives discussed it with women, or how appropriate stakeholders thought it was. These factors may have influenced effectiveness and variations in effectiveness between sub-groups.[31]

Conclusions

Although the introduction of the HiPG was associated with an immediate clinical deterioration in timing of attendance for first antenatal care, it was also associated with a longer term trend towards improvement in timing. By 21 months post-implementation, there was a decrease in almost five days in mean gestational age at booking compared to what would have been expected without implementation. Withdrawal of the HiPG was associated with deteriorations in timing of booking. By 24 months post-withdrawal there was an increase in 14 days in mean gestational age at booking compared to what would have been expected without withdrawal. Neither the introduction nor withdrawal of the HiPG was associated with a change in proportion of babies who were SGA. There was no evidence that associations between introduction or withdrawal of the HiPG and outcomes varied by maternal parity. Introduction of the HiPG was associated with greater long-term benefits on timing at booking in older women suggesting older women were most responsive to the intervention. The initial deterioration in timing of attendance for first antenatal care was least pronounced in those living in the most deprived circumstances suggesting those living in the most deprived circumstances were most responsive to the intervention. Future research should explore the effects of incentives offered at different times in pregnancy and of differing values; and how stakeholders view such incentives.

For peer review only - http://bmjopen.bmj.com/site/about/guidelines.xhtml

295

For peer review only

Funding

This work is produced under the terms of a Career Development Fellowship research training fellowship issued by the National Institute of Health Research [grant number CDF-2011-04-001] to JA. The views expressed are those of the authors and not necessarily those of the NHS, The National Institute for Health Research or the Department of Health. JA is supported by the Centre for Diet and Activity Research (CEDAR), a UKCRC Public Health Research Centre of Excellence. Funding from the British Heart Foundation, Cancer Research UK, Economic and Social Research Council, Medical Research Council, the National Institute for Health Research, and the Wellcome Trust, under the auspices of the UK Clinical Research Collaboration, is gratefully acknowledged [grant number MR/K023187/1].

Role of the funding sources

The funders played no role in any aspect of study design, data analysis, writing or the decision to submit. All authors had full access to all the data and had final responsibility for the decision to submit. No authors were paid by a pharmaceutical company or other agency to write this article. All authors are independent from the funders.

For peer review only

Competing interests

All authors have completed the ICMJE uniform disclosure form at www.icmje.org/coi_disclosure.pdf and declare: JA had financial support from the National Institute for Health Research and the Centre for Diet and Activity Research (as described under ‘funding’) for the submitted work; no financial relationships with any organisations that might have an interest in the submitted work in the previous three years; no other relationships or activities that could appear to have influenced the submitted work.

Contributions

JA conducted the literature searches, obtained the data, conducted the data analysis and led writing. ZvdW, SR, and JR contributed to study design, development of the analysis plan, interpretation of the data and critically reviewed previous versions of the final manuscript. JA will act as guarantor.

For peer review only - http://bmjopen.bmj.com/site/about/guidelines.xhtml

Research ethics

Ethics approval was granted by the East of England Norfolk NHS Research Ethics Committee (12/EE/0386). The routine hospital data used in this study was anonymised before transfer to the research team and the ethics committee determined that explicit patient consent was not required.

Data Sharing

No additional data available.

For peer review only

References

1. Giles E, Robalino S, McColl E, et al. Systematic review, meta-analysis and meta-regression of the effectiveness of financial incentives for encouraging healthy behaviours. *PLoS ONE* 2014;9 doi: 10.1371/journal.pone.0090347

2. Mantzari E, Vogt F, Shemilt I, et al. Personal financial incentives for changing habitual health-related behaviors: A systematic review and meta-analysis. *Prev Med* 2015;75:75-85. doi: 10.1016/j.ypmed.2015.03.001 [published Online First: 2015/04/07]

3. HM Revenue & Customs, The Royal College of Midwives. Health in Pregnancy Grant: Frequently Asked Questions 2009 [Available from: http://www.direct.gov.uk/en/groups/dg_digitalassets/@dg/@en/documents/digitalasset/dg_173401.pdf accessed 25 March 2010.

4. EURO-PERISTAT project, SCPE, EUROCAT, et al. European Perinatal Health Report. Paris, 2008.

5. VanderWeele TJ, Lantos JD, Siddique J, et al. A comparison of four prenatal care indices in birth outcome models: Comparable results for predicting small-for-gestational-age outcome but different results for preterm birth or infant mortality. *J Clin Epidemiol* 2009;62(4):438-45.

6. Heaman M, Newburn-Cook C, Green C, et al. Inadequate prenatal care and its association with adverse pregnancy outcomes: A comparison of indices. *BMC Pregnancy and Childbirth* 2008;8(1):15.

7. Lin C, Chen C, Chen P, et al. Risks and causes of mortality among low-birthweight infants in childhood and adolescence. *Paediatr Perinat Epidemiol* 2007;21(5):465-72.

8. Mikkola K, Ritari N, Tommiska V, et al. Neurodevelopmental outcome at 5 years of age of a national cohort of extremely low birth weight infants who were born in 1996-1997. *Pediatrics* 2005;116(6):1391-400. doi: 10.1542/peds.2005-0171 [published Online First: 2005/12/03]

9. Tommiska V, Heinonen K, Ikonen S, et al. A national short-term follow-Up study of extremely low birth weight infants born in Finland in 1996-1997. *Pediatrics* 2001;107(1):E2. [published Online First: 2001/01/03]

10. National Collaborating Centre for Women's and Children's Health. Antenatal care: Routine care for the healthy pregnant woman. London: National Institute for Health and Clinical Excellence, 2008.

11. Rowe RE, Garcia J. Social class, ethnicity and attendance for antenatal care in the United Kingdom: a systematic review. *J Public Health* 2003;25(2):113-19. doi: 10.1093/pubmed/fdg025

12. Sutherland K, Christianson JB, Leatherman S. Impact of Targeted Financial Incentives on Personal Health Behavior: A Review of the Literature. *Med Care Res Rev* 2008;65(6_suppl):36S-78. doi: 10.1177/1077558708324235

13. Marteau T, Ashcroft R, Oliver A. Using financial incentives to achieve healthy behaviour. *BMJ* 2009;338 doi: 10.1136/bmj.b1415

14. Jochelson K. Paying the Patient: improving health using financial incentives. London: King's Fund, 2007.

15. Forde I, Zeuner D. Financial incentives to promote social mobility. *BMJ* 2009;339:b3219. doi: 10.1136/bmj.b3219

For peer review only - http://bmjopen.bmj.com/site/about/guidelines.xhtml

16. Till SR, Everetts D, Haas DM. Incentives for increasing prenatal care use by women in order to improve maternal and neonatal outcomes. *Cochrane Database Syst Rev* 2015(12) doi: 10.1002/14651858.CD009916.pub2

17. Melnikow J, Paliescheskey M, Stewart G. Effect of a transportation incentive on compliance with the first prenatal appointment: a randomized trial. *Obstet Gynecol* 1997;89:1023-7.

18. Rosenthal M, Li Z, Robertson A, et al. Impact of financial incentive for prenatal care on birth outcomes and spending. *HSR: Health Services Research* 2009;44:1465-79.

19. Leyland A, Ouedraogo S, Gray R, et al. Evaluating health in pregnancy grants in Scotland: a natural experiment. *J Epidemiol Community Health* 2016;70(S1):A47-8.

20. Giles E, McColl E, Sniehotta F, et al. Acceptability of financial incentives and penalties for encouraging uptake of healthy behaviours: focus groups. *BMC Public Health* 2015;15 doi: 10.1186/s12889-015-1409-y

21. Giles EL, Sniehotta FF, McColl E, et al. Acceptability of financial incentives for health behaviour change to public health policymakers: a qualitative study. *BMC Public Health* 2016;16:989. doi: 10.1186/s12889-016-3646-0 [published Online First: 2016/09/17]

22. Lorenc T, Petticrew M, Welch V, et al. What types of interventions generate inequalities? Evidence from systematic reviews. *J Epidemiol Community Health* 2012;67(2):190-3. doi: 10.1136/jech-2012-201257

23. Craig P, Cooper C, Gunnell D, et al. Using natural experiments to evaluate population health interventions: guidance for producers and users of evidence: Medical Research Council, 2011.

24. Penfold RB, Zhang F. Use of interrupted time series analysis in evaluating health care quality improvements. *Academic Pediatrics* 2013;13(6 Suppl):S38-44. doi: 10.1016/j.acap.2013.08.002 [published Online First: 2013/12/07]

25. Bonellie S, Chalmers J, Gray R, et al. Centile charts for birthweight for gestational age for Scottish singleton births. *BMC Pregnancy & Childbirth* 2008;8:5. doi: 10.1186/1471-2393-8-5 [published Online First: 2008/02/27]

26. Department of Communities and Local Government. The English Indices of Deprivation 2010 2011 [Available from: http://www.communities.gov.uk/publications/corporate/statistics/indices201015 October 2012.

27. Zhang F, Wagner AK, Soumerai SB, et al. Methods for estimating confidence intervals in interrupted time series analyses of health interventions. *J Clin Epidemiol* 2009;62(2):10.1016/j.jclinepi.2008.08.007. doi: 10.1016/j.jclinepi.2008.08.007

28. Jandoc R, Burden AM, Mamdani M, et al. Interrupted time series analysis in drug utilization research is increasing: systematic review and recommendations. *J Clin Epidemiol* 2015;68(8):950-6. doi: 10.1016/j.jclinepi.2014.12.018 [published Online First: 2015/04/22]

29. Adams J, Giles E, McColl E, et al. Carrots, sticks, and health behaviours: a framework for documenting the complexity of financial incentive interventions to change health behaviours. *Health Psychology Review* 2014;8(3):286-95.

30. Giles E, Robalino S, Sniehotta F, et al. Acceptability of financial incentives for encouraging uptake of healthy behaviours: A critical review using systematic methods. *Prev Med* 2015;73 doi: 10.1016/j.ypmed.2014.12.029

For peer review only - http://bmjopen.bmj.com/site/about/guidelines.xhtml

31. Giles EL, Adams JM. Capturing public opinion on public health topics: a comparison of experiences from a systematic review, focus group study, and analysis of online, user-generated content. *Frontiers in Public Health* 2015;3 doi: 10.3389/fpubh.2015.00200

For peer review only

Table 1. Characteristics of those included and excluded from the analytical cohort

		Before HiPG availability (January 2003 – March 2009)		During HiPG availability (April 2009 – December 2010)		After HiPG availability (January 2011 – December 2013)		Full study period (January 2003 – December 2013)	
Variable	Level	Included[1]	Excluded[2]	Included[1]	Excluded[2]	Included[1]	Excluded[2]	Included[1]	Excluded[2]
N(%)		18 744	2816	6126	862	9719	1304	34 589	4982
Maternal age, n(%)	<25 years	6359 (33.9)	1327 (47.1)	2039 (33.3)	374 (43.4)	2836 (29.2)	523 (40.1)	11 234 (32.5)	2224 (44.6)
	25-34 years	9684 (51.7)	1208 (42.9)	3272 (53.4)	405 (47.0)	5577 (57.4)	651 (49.9)	18 533 (53.6)	2264 (45.4)
	35 years +	2701 (14.4)	281 (10.0)	815 (13.3)	83 (9.6)	1306 (13.4)	130 (10.0)	4822 (13.9)	494 (9.9)
	Data not available, n(%)	0	0[3]	0	0[3]	0	0[3]	0	0[3]
Parity, n(%)	0	8077 (43.1)	1288 (45.7)	2670 (43.6)	378 (43.9)	3944 (40.6)	585 (44.9)	14 691 (42.5)	2251 (45.2)
	1+	10 667 (56.9)	1528 (54.3)	3456 (56.4)	484 (56.2)	5775 (59.4)	719 (55.1)	19 898 (57.5)	2731 (54.8)
	Data not available, n(%)	0	0[3]	0	0	0	0[3]	0	0[3]
Index of multiple deprivation group, n(%)	Most deprived	10 820 (57.7)	1777 (63.1)	3566 (58.2)	547 (63.5)	5821 (59.9)	794 (60.9)	20 207 (58.4)	3118 (62.6)
	Moderately deprived	4213 (22.5)	490 (17.4)	1330 (21.7)	160 (18.6)	2230 (22.9)	220 (16.9)	7773 (22.5)	870 (17.5)
	Least deprived	3711 (19.8)	361 (12.8)	1230 (20.1)	92 (10.7)	1668 (17.2)	128 (9.8)	6609 (19.1)	581 (11.7)
	Data not available, n(%)	0	188 (6.7)[3]	0	63 (7.3)[3]	0	162 (12.4)[3]	0	413 (8.3)[3]
Study outcomes	Mean (SD) gestational age at booking, days	76.7 (35.8)	106.2 (45.5)[4]	77.8 (41.3)	99.3 (39.8)[4]	71.8 (31.8)	95.0 (39.1)[4]	75.6 (35.8)	102.0 (43.2)[4]
	Booked by 10 weeks, n(%)	10 261 (54.7)	442 (17.0)[3]	3540 (57.8)	152 (18.8)[3]	6127 (63.0)	283 (23.9)[3]	19 928 (57.6)	877 (19.1)[3]
	Booked by 18 weeks, n(%)	17 579 (93.8)	2012 (77.7)[)3]	5675 (92.6)	676 (83.7)[3]	9307 (95.8)	1022 (86.3)[3]	32 561 (94.1)	3719 (81.0)[3]
	Booked by 25 weeks, n(%)	18 098 (96.6)	2397 (92.1)[3]	5846 (95.4)	766 (94.8)	9477 (97.5)	1129 (95.4)[3]	33 421 (96.6)	4292 (93.4)[4=3]
	Small for gestational age, n(%)	2346 (12.5)	17 (8.3)	743 (12.1)	7 (11.5)	1163 (12.0)	25 (14.9)	4252 (12.3)	49 (11.3)
	Data not available on time at booking, n(%)	0	214 (7.6)	0	54 (6.3)	0	120 (9.2)	0	388 (7.8)
	Data not available on birth weight, n(%)	0	2611 (92.7)	0	801 (92.9)	0	1136 (87.1)	0	4548 (91.3)

For peer review only - http://bmjopen.bmj.com/site/about/guidelines.xhtml

For peer review only

[1]Women who delivered at the study hospital, were known to have reached the 25th week of gestation between 1st January 2003 and 31st December 2013 and had available data on all variables of interest; [2]Women who delivered at the study hospital, were known to have reached the 25th week of gestation between 1st January 2003 and 31st December 2013 and did not have available data on all variables of interest; [3]chi-squared test indicates difference in distribution of levels between included and excluded at a level of $p<0.05$; [4]t-test indicates difference in means between included and excluded at a level of $p<0.05$

HiPG: Health in Pregnancy Grant

For peer review only - http://bmjopen.bmj.com/site/about/guidelines.xhtml

Table 2. Summary of interrupted time series models of the associations between the introduction and withdrawal of the Health in Pregnancy Grant and outcomes of interest, coefficients (95% CI)

Model variable	Mean gestational age at booking (days)	Proportion booking by 10 weeks	Proportion booking by 18 weeks	Proportion booking by 25 weeks	Proportion of babies SGA
Time (months)	0.02 (-0.01 to 0.05)	0.0003[1] (-0.005 to 0.007)	**-0.0002 (-0.0003 to -0.00003)**[2]	-0.0001 (-0.0002 to 0.00005)	-0.00003 (-0.0003 to 0.0002)
Level change at introduction	**5.29 (2.47 to 8.11)**	-0.04 (-0.10 to 0.01)	**-0.03 (-0.04 to -0.02)**	**-0.03 (-0.05 to -0.02)**	0.005 (-0.02 to 0.03)
Trend change at introduction	**-0.50 (-0.70 to -0.30)**	**0.005 (0.001 to 0.009)**	**0.003 (0.002 to 0.004)**	**0.003 (0.002 to 0.003)**	-0.001 (-0.002 to 0.001)
Level change at withdrawal	1.37 (-1.63 to 4.37)	-0.03 (-0.08 to 0.02)	0.001 (-0.01 to 0.02)	-0.01 (-0.02 to 0.004)	0.01 (-0.01 to 0.03)
Trend change at withdrawal	**0.35 (0.13 to 0.57)**	-0.003 (-0.008 to 0.002)	**-0.002 (-0.003 to -0.001)**	**-0.002 (-0.003 to -0.001)**	0.00 (-0.001 to 0.002)

[1]Values are given to two decimal places or, for values <0.1, one significant figure. Values <0.001 are shown as 0.00; [2]Bold indicates where 95% confidence intervals do not cross 0.

For peer review only - http://bmjopen.bmj.com/site/about/guidelines.xhtml

417 **Table 3. Predicted effects (95% CI) of introduction and withdrawal of the Health in Pregnancy Grant at 24 months after each event**

Outcome	21 months after introduction		24 months after withdrawal	
	Absolute change	Relative change (%)	Absolute change	Relative change (%)
Mean gestational age at booking (days)	**-4.8 (-8.2 to -2.3)**[1]	**-6.2 (-10.5 to -3.0)**	**14.0 (2.8 to 16.8)**	**25.2 (2.1 to 33.2)**
Proportion booking by 10 weeks	0.06 (-0.02 to 12.5)	10.3 (-4.2 to 22.6)	-0.14 (-0.24 to 0.03)	-17.4 (-26.8 to 1.3)
Proportion booking by 18 weeks	**0.02 (0.01 to 0.04)**	**2.2 (1.2 to 3.9)**	**-0.08 (-0.08 to -0.02)**	**-7.6 (-7.9 to -2.2)**
Proportion booking by 25 weeks	**0.02 (0.01 to 0.03)**	**1.9 (0.6 to 3.5)**	**-0.09 (-0.09 to -0.03)**	**-8.3 (-8.6 to -3.1)**
Proportion of babies small for gestational age	-0.01 (-0.03 to 0.01)	-8.1 (-25.9 to 10.8)	0.03 (-0.03 to 0.08)	29.8 (-57.4 to 104.9)

418 [1]Bold indicates where 95% confidence intervals do not cross 0

For peer review only - http://bmjopen.bmj.com/site/about/guidelines.xhtml

Table 4. Summary of interactions between parity and the introduction and withdrawal of the Health in Pregnancy Grant, model coefficients (95% CI),

Model variable	Mean gestational age booking	Proportion booking by 10 weeks	Proportion booking by 18 weeks	Proportion booking by 25 weeks	Proportion of babies SGA
Time	0.03 (-0.01 to 0.08)	-0.0002 (-0.001 to 0.0004)[1]	-0.0002 (-0.0004 to 0.00001)	-0.0001 (-0.0003 to 0.00007)	-0.00002 (-0.0003 to 0.0003)
Parity >0 vs 0	0.09 (-2.54 to 2.72)	**-0.04 (-0.09 to -0.003)**[2]	0.008 (-0.006 to 0.02)	0.01 (-0.001 to 0.02)	-0.004 (-0.02 to 0.02)
Parity * time	-0.02 (-0.08 to 0.04)	0.0005 (-0.0005 to 0.001)	0.0002 (-0.002 to 0.002)	0.00005 (-0.0002 to 0.0003)	-0.00002 (-0.0005 to 0.0004)
Level change at introduction	**5.57 (1.62 to 9.51)**	0.01 (-0.05 to 0.07)	**-0.04 (-0.06 to -0.01)**	**-0.04 (-0.05 to -0.02)**	0.01 (-0.02 to 0.04)
Trend change at introduction	**-0.50 (-0.78 to -0.21)**	0.003 (-0.001 to 0.008)	**0.002 (0.001 to 0.004)**	**0.003 (0.001 to 0.004)**	-0.001 (-0.004 to 0.001)
Parity * level change at introduction	-0.61 (-6.19 to 4.97)	-0.04 (-0.13 to 0.04)	0.007 (-0.02 to 0.04)	0.007 (-0.02 to 0.03)	-0.01 (-0.06 to 0.03)
Parity * trend change at introduction	0.01 (-0.39 to 0.41)	0.0006 (-0.006 to 0.007)	-0.001 (-0.002 to 0.002)	-0.0002 (-0.002 to 0.002)	0.002 (-0.002 to 0.005)
Level change at withdrawal	1.03 (-3.16 to 5.22)	-0.02 (-0.08 to 0.05)	0.005 (-0.02 to 0.03)	-0.01 (-0.03 to 0.001)	0.02 (-0.04 to 0.02)
Trend change at withdrawal	0.30 (-0.01 to 0.61)	-0.002 (-0.006 to 0.003)	**-0.002 (-0.003 to -0.00)**	-0.002 (-0.003 to 0.001)	0.001 (-0.002 to 0.003)
Parity * level change at withdrawal	-0.13 (-5.80 to 6.06)	0.006 (-0.09 to 0.10)	-0.003 (-0.04 to 0.03)	0.001 (-0.03 to 0.03)	-0.03 (-0.07 to 0.02)
Parity * trend change at withdrawal	0.08 (-0.36 to 0.52)	-0.001 (-0.008 to 0.006)	-0.001 (-0.003 to 0.002)	-0.00 (-0.002 to 0.002)	-0.001 (-0.004 to 0.003)

[1]Values are given to two decimal places or, for values <0.1, one significant figure. Values <0.001 are shown as 0.00; [2]Bold indicates where 95% confidence intervals do not cross 0.

For peer review only

422 **Table 5. Summary of interactions between maternal age and the introduction and withdrawal of the Health in Pregnancy Grant, model coefficients (95% CI)**

	Mean gestational age booking	Proportion booking by 10 weeks	Proportion booking by 18 weeks	Proportion booking by 25 weeks	Proportion of babies SGA
Time	-0.01 (-0.06 to 0.05)	0.00[1] (-0.001 to 0.002)	-0.00 (-0.00 to 0.00)	-0.00 (-0.00 to 0.00)	-0.00 (-0.00 to 0.00)
Age group	**-3.41 (-5.39 to -1.42)**[2]	**0.05 (0.002 to 0.11)**	**0.01 (0.004 to 0.02)**	0.00 (-0.006 to 0.007)	**-0.04 (-0.05 to -0.03)**
Age * time	0.03 (-0.01 to 0.08)	-0.00 (-0.001 to 0.001)	-0.00 (-0.00 to 0.00)	-0.00 (-0.00 to 0.00)	0.00 (-0.00 to 0.001)
Level change at introduction	3.02 (-0.87 to 6.90)	-0.04 (-0.10 to 0.02)	-0.03 (-0.05 to -0.001)	**-0.02 (-0.04 to -0.004)**	-0.002 (-0.04 to 0.03)
Trend change at introduction	-0.25 (-0.53 to 0.04)	0.002 (-0.003 to 0.008)	0.002 (-0.00 to 0.004)	**0.002 (0.00 to 0.003)**	0.00 (-0.002 to 0.003)
Age * level change at introduction	2.15 (-0.86 to 5.16)	-0.01 (-0.06 to 0.04)	-0.003 (-0.02 to 0.02)	-0.005 (-0.02 to 0.001)	0.005 (-0.02 to 0.03)
Age * trend change at introduction	**-0.26 (-0.48 to -0.04)**	0.001 (-0.003 to 0.005)	0.001 (-0.00 to 0.002)	0.001 (-0.00 to 0.002)	-0.001 (-0.003 to 0.001)
Level change at withdrawal	0.57 (-3.45 to 4.59)	-0.04 (-0.10 to 0.02)	0.01 (-0.02 to 0.04)	-0.006 (-0.03 to 0.02)	0.007 (-0.03 to 0.05)
Trend change at withdrawal	0.08 (-0.24 to 0.40)	-0.00 (-0.006 to 0.005)	-0.001 (-0.003 to 0.001)	-0.001 (-0.003 to 0.00)	-0.001 (-0.00 to 0.002)
Age * level change at withdrawal	1.23 (-1.88 to 4.34)	0.01 (-0.04 to 0.06)	-0.01 (-0.04 to 0.01)	-0.008 (-0.02 to 0.008)	0.002 (-0.03 to 0.03)
Age * trend change at withdrawal	**0.27 (0.02 to 0.52)**	-0.002 (-0.007 to 0.002)	-0.00 (-0.002 to 0.001)	-0.001 (-0.002 to 0.00)	0.001 (-0.001 to 0.003)

423 [1]Values are given to two decimal places or, for values <0.1, one significant figure. Values <0.001 are shown as 0.00; [2]Bold indicates where 95% confidence
424 intervals do not cross 0.

For peer review only

For peer review only - http://bmjopen.bmj.com/site/about/guidelines.xhtml

Table 6. Summary of interactions between Index of Multiple Deprivation group and the introduction and withdrawal of the Health in Pregnancy Grant, model coefficients (95% CI)

	Mean gestational age booking	Proportion booking by 10 weeks	Proportion booking by 18 weeks	Proportion booking by 25 weeks	Proportion of babies SGA
Time	**0.11 (0.03 to 0.19)**[1]	-0.001 (-0.003 to 0.001)	**-0.001 (-0.001 to -0.001)**	-0.00 (-0.00 to 0.00)[2]	0.00 (-0.00 to 0.00)
Deprivation tertile	**4.53 (2.90 to 6.15)**	**-0.09 (-0.13 to -0.05)**	**-0.02 (-0.03 to -0.001)**	-0.005 (-0.01 to 0.003)	**0.04 (0.03 to 0.05)**
Deprivation * time	**-0.04 (-0.07 to -0.00)**	0.00 (-0.00 to 0.001)	0.00 (-0.00 to 0.00)	0.00 (-0.00 to 0.00)	-0.00 (-0.00 to 0.00)
Level change at introduction	**17.18 (9.55 to 24.81)**	-0.06 (-0.17 to 0.06)	**-0.09 (-0.14 to -0.05)**	**-0.10 (-0.13 to -0.06)**	0.01 (-0.05 to 0.07)
Trend change at introduction	**-1.04 (-1.59 to -0.49)**	0.005 (-0.003 to 0.01)	**0.004 (0.001 to 0.007)**	**0.005 (0.002 to 0.008)**	-0.00 (-0.005 to 0.003)
Deprivation * level change at introduction	**-5.02 (-8.56 to -1.49)**	0.007 (-0.05 to 0.06)	**0.03 (0.004 to 0.05)**	**0.03 (0.01 to 0.04)**	-0.001 (-0.03 to 0.03)
Deprivation * trend change at introduction	0.22 (-0.03 to 0.47)	-0.00 (-0.004 to 0.004)	-0.001 (-0.002 to 0.001)	-0.001 (-0.002 to -0.00)	0.00 (-0.002 to 0.002)
Level change at withdrawal	6.47 (-1.68 to 14.62)	-0.06 (-0.18 to 0.06)	-0.008 (-0.06 to 0.04)	-0.03 (-0.07 to 0.01)	0.02 (-0.04 to 0.08)
Trend change at withdrawal	0.59 (-0.005 to 1.18)	-0.002 (-0.01 to 0.01)	-0.002 (-0.006 to 0.001)	**-0.003 (-0.006 to -0.00)**	-0.001 (-0.006 to 0.003)
Deprivation * level change at withdrawal	-2.10 (-5.88 to 1.67)	0.01 (-0.04 to 0.07)	0.004 (-0.02 to 0.03)	0.008 (-0.01 to 0.03)	-0.003 (-0.03 to 0.03)
Deprivation * trend change at withdrawal	-0.10 (-0.37 to 0.18)	-0.00 (-0.005 to 0.005)	0.00 (-0.002 to 0.002)	0.00 (-0.001 to 0.002)	0.00 (-0.001 to 0.003)

[1]Bold indicates where 95% confidence intervals do not cross 0; [2]values are given to two decimal places or, for values <0.1, one significant figure. Values <0.001 are shown as 0.00.

For peer review only

For peer review only - http://bmjopen.bmj.com/site/about/guidelines.xhtml

Figure legends

Figure 1. Summary of interrupted time series model of the introduction and withdrawal of the Health in Pregnancy Grant on mean gestational age at booking (days)

Figure 2. Summary of interrupted time series model of the introduction and withdrawal of the Health in Pregnancy Grant on proportion booking before 10 weeks gestation

Figure 3. Summary of interrupted time series model of the introduction and withdrawal of the Health in Pregnancy Grant on proportion booking before 18 weeks gestation

Figure 4. Summary of interrupted time series model of the introduction and withdrawal of the Health in Pregnancy Grant on proportion booking before 25 weeks gestation

Figure 5. Summary of interrupted time series model of the introduction and withdrawal of the Health in Pregnancy Grant on proportion of babies small for gestational age

Figure 6. Summary of interrupted time series model, interaction between maternal age group and the introduction and withdrawal of the Health in Pregnancy Grant on mean gestational age at booking (days)

Figure 7. Summary of interrupted time series model, interactions between Index of Multiple Deprivation group and the introduction and withdrawal of the Health in Pregnancy Grant on mean gestational age at booking (days)

Figure 8. Summary of interrupted time series model, interactions between Index of Multiple Deprivation group and the introduction and withdrawal of the Health in Pregnancy Grant on proportion booking before 18 weeks gestation

Figure 9. Summary of interrupted time series model, interactions between Index of Multiple Deprivation group and the introduction and withdrawal of the Health in Pregnancy Grant on proportion booking before 25 weeks gestation

For peer review only

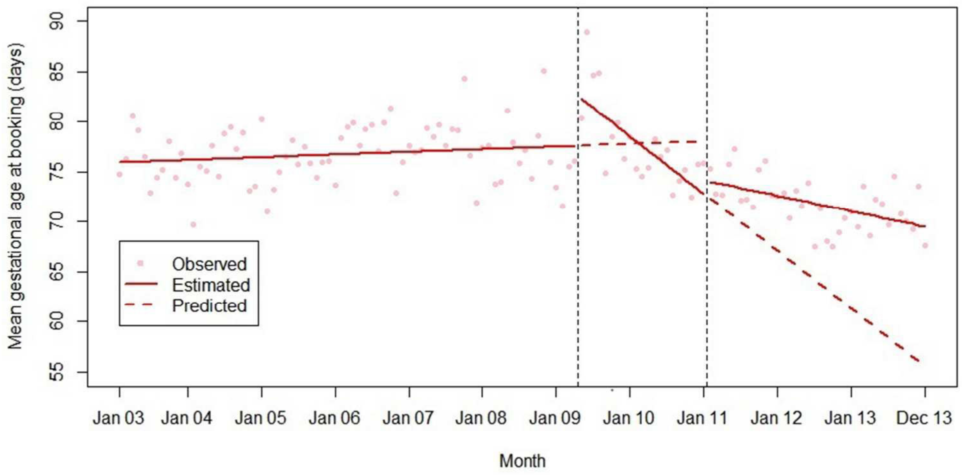

Summary of interrupted time series model of the introduction and withdrawal of the Health in Pregnancy Grant on mean gestational age at booking (days)

76x44mm (300 x 300 DPI)

For peer review only

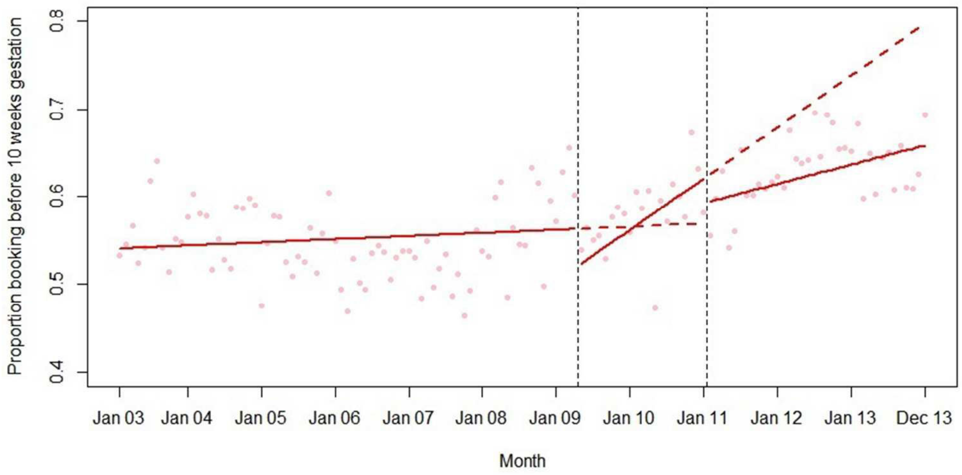

Summary of interrupted time series model of the introduction and withdrawal of the Health in Pregnancy Grant on proportion booking before 10 weeks gestation

76x44mm (300 x 300 DPI)

For peer review only

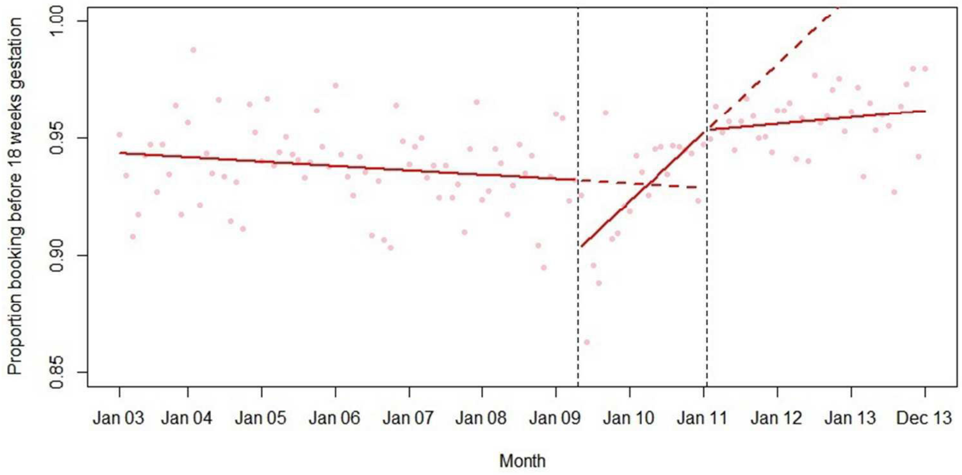

Summary of interrupted time series model of the introduction and withdrawal of the Health in Pregnancy Grant on proportion booking before 18 weeks gestation

76x44mm (300 x 300 DPI)

For peer review only - http://bmjopen.bmj.com/site/about/guidelines.xhtml

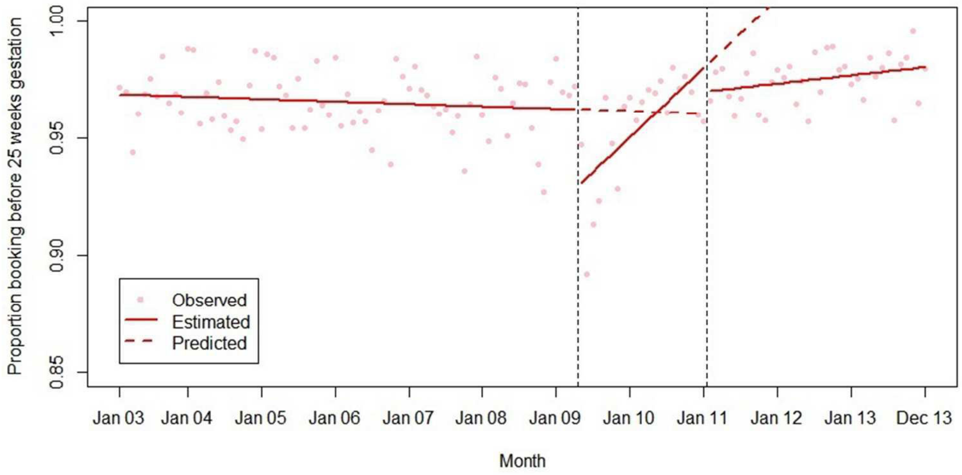

Summary of interrupted time series model of the introduction and withdrawal of the Health in Pregnancy Grant on proportion booking before 25 weeks gestation

76x44mm (300 x 300 DPI)

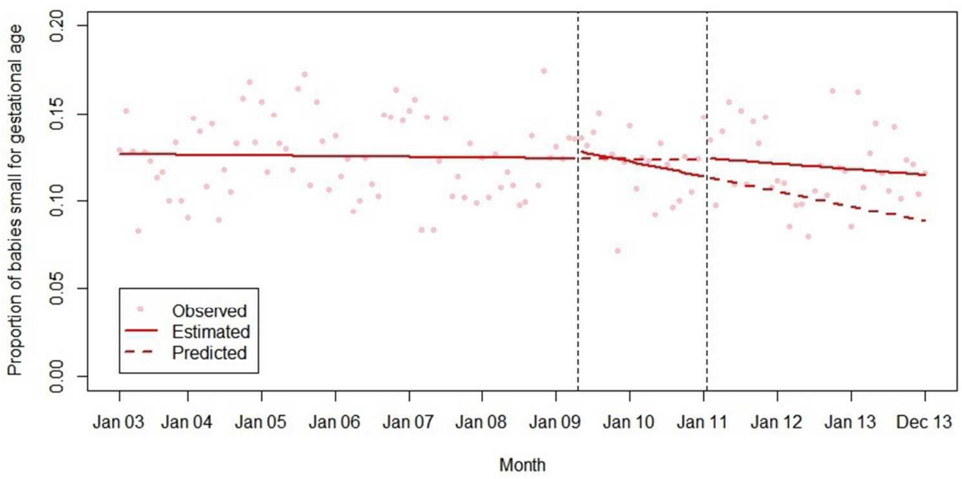

Summary of interrupted time series model of the introduction and withdrawal of the Health in Pregnancy Grant on proportion of babies small for gestational age

76x44mm (300 x 300 DPI)

For peer review only

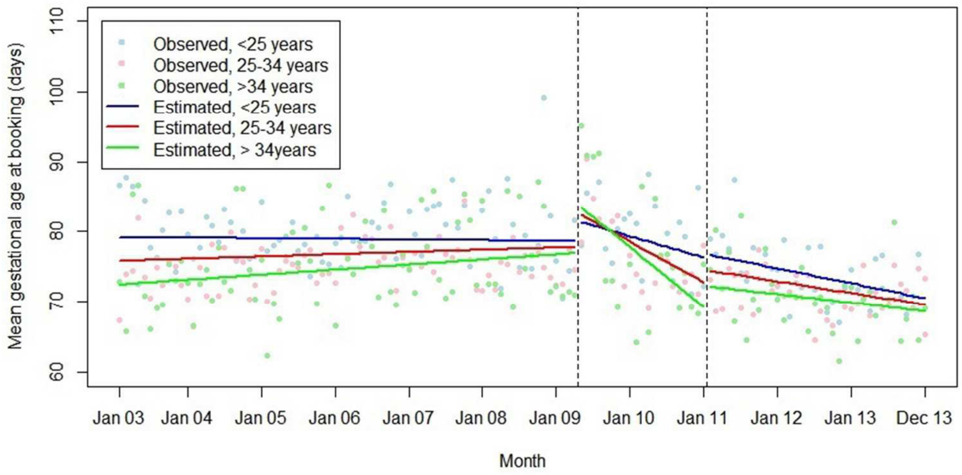

Summary of interrupted time series model, interaction between maternal age group and the introduction and withdrawal of the Health in Pregnancy Grant on mean gestational age at booking (days)

76x44mm (300 x 300 DPI)

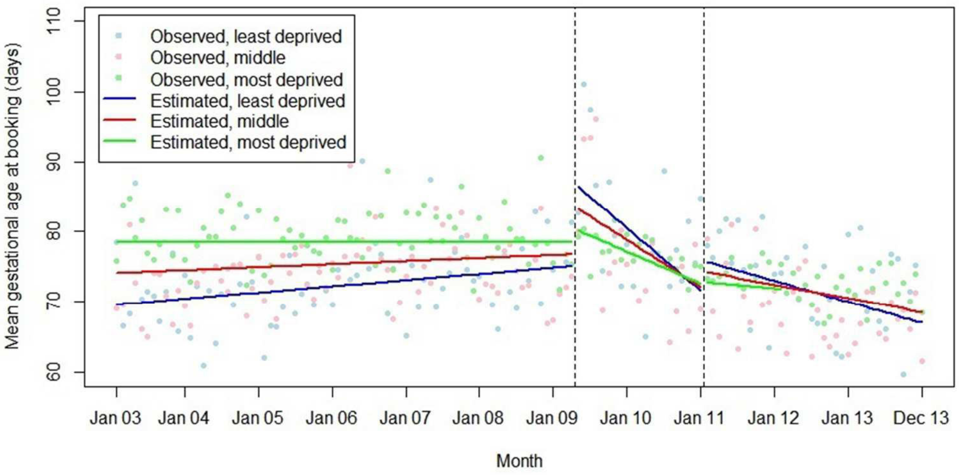

Summary of interrupted time series model, interactions between Index of Multiple Deprivation group and the introduction and withdrawal of the Health in Pregnancy Grant on mean gestational age at booking (days)

76x44mm (300 x 300 DPI)

For peer review only

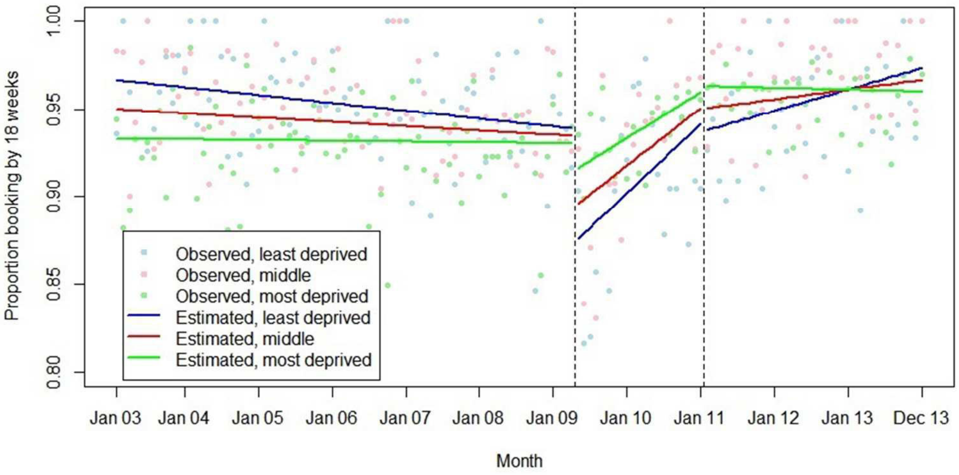

Summary of interrupted time series model, interactions between Index of Multiple Deprivation group and the introduction and withdrawal of the Health in Pregnancy Grant on proportion booking before 18 weeks gestation

76x44mm (300 x 300 DPI)

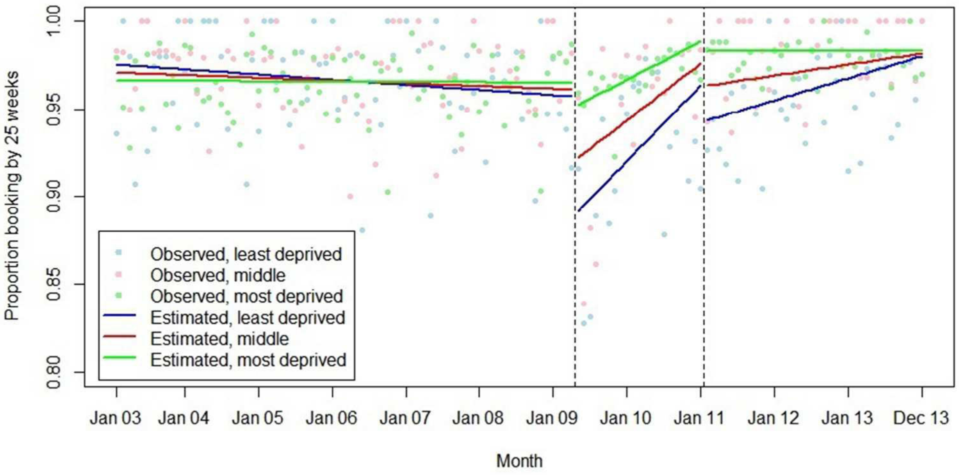

Summary of interrupted time series model, interactions between Index of Multiple Deprivation group and the introduction and withdrawal of the Health in Pregnancy Grant on proportion booking before 25 weeks gestation

76x44mm (300 x 300 DPI)

STROBE Statement—checklist of items that should be included in reports of observational studies

	Item No	Recommendation	Page/line ref
**Title and abstract**	1	(*a*) Indicate the study's design with a commonly used term in the title or the abstract	p1, lines 2-3
		(*b*) Provide in the abstract an informative and balanced summary of what was done and what was found	p2, lines 17-37
**Introduction**			
Background/rationale	2	Explain the scientific background and rationale for the investigation being reported	p4-5, lines 58-84
Objectives	3	State specific objectives, including any prespecified hypotheses	p5, lines 85-88
**Methods**			
Study design	4	Present key elements of study design early in the paper	p5, line 90
Setting	5	Describe the setting, locations, and relevant dates, including periods of recruitment, exposure, follow-up, and data collection	p5-6, lines 92-106
Participants	6	(*a*) *Cohort study*—Give the eligibility criteria, and the sources and methods of selection of participants. Describe methods of follow-up<br>*Case-control study*—Give the eligibility criteria, and the sources and methods of case ascertainment and control selection. Give the rationale for the choice of cases and controls<br>*Cross-sectional study*—Give the eligibility criteria, and the sources and methods of selection of participants	p5-6, lines 92-106
		(*b*) *Cohort study*—For matched studies, give matching criteria and number of exposed and unexposed<br>*Case-control study*—For matched studies, give matching criteria and the number of controls per case	No matching
Variables	7	Clearly define all outcomes, exposures, predictors, potential confounders, and effect modifiers. Give diagnostic criteria, if applicable	Exposures – p5, lines 95-102<br>Outcomes – p6, lines 106-114<br>Potential effect modifiers – p6, lines 116-121
Data sources/ measurement	8*	For each variable of interest, give sources of data and details of methods of assessment (measurement). Describe comparability of assessment methods if there is more than one group	p5-6, lines 92-121
Bias	9	Describe any efforts to address potential sources of bias	p6-7, lines 123-127; p10-11, lines 216-220
Study size	10	Explain how the study size was arrived at	p5, lines 95-101
Quantitative variables	11	Explain how quantitative variables were handled in the analyses. If applicable, describe which groupings were chosen and why	p6-7, lines 114-141

For peer review only

For peer review only - http://bmjopen.bmj.com/site/about/guidelines.xhtml

Statistical methods	12	(*a*) Describe all statistical methods, including those used to control for confounding	p7, lines 127-141
		(*b*) Describe any methods used to examine subgroups and interactions	p7, lines 139-140
		(*c*) Explain how missing data were addressed	p6-7, lines 121-125
		(*d*) *Cohort study*—If applicable, explain how loss to follow-up was addressed<br>*Case-control study*—If applicable, explain how matching of cases and controls was addressed<br>*Cross-sectional study*—If applicable, describe analytical methods taking account of sampling strategy	Not applicable
		(*e*) Describe any sensitivity analyses	None – see p10-11, lines 214-218
**Results**			
Participants	13*	(a) Report numbers of individuals at each stage of study—eg numbers potentially eligible, examined for eligibility, confirmed eligible, included in the study, completing follow-up, and analysed	Table 1
		(b) Give reasons for non-participation at each stage	Table 1
		(c) Consider use of a flow diagram	Not considered helpful
Descriptive data	14*	(a) Give characteristics of study participants (eg demographic, clinical, social) and information on exposures and potential confounders	Table 1
		(b) Indicate number of participants with missing data for each variable of interest	Table 1
		(c) *Cohort study*—Summarise follow-up time (eg, average and total amount)	Not applicable
Outcome data	15*	*Cohort study*—Report numbers of outcome events or summary measures over time	Not applicable
		Case-control study—Report numbers in each exposure category, or summary measures of exposure	Not applicable
		Cross-sectional study—Report numbers of outcome events or summary measures	Table 1
Main results	16	(*a*) Give unadjusted estimates and, if applicable, confounder-adjusted estimates and their precision (eg, 95% confidence interval). Make clear which confounders were adjusted for and why they were included	Tables 2-6
		(*b*) Report category boundaries when continuous variables were categorized	Table 1
		(*c*) If relevant, consider translating estimates of relative risk into absolute risk for a meaningful time period	Table 3
Other analyses	17	Report other analyses done—eg analyses of subgroups and interactions, and sensitivity analyses	Tables 4-6
**Discussion**			
Key results	18	Summarise key results with reference to study objectives	p9-10, lines 183-195
Limitations	19	Discuss limitations of the study, taking into account sources of potential bias or imprecision. Discuss both direction and magnitude of any potential bias	p10, lines 197-212
Interpretation	20	Give a cautious overall interpretation of results considering objectives, limitations, multiplicity of analyses, results from similar studies, and other relevant evidence	p10-12, lines 214-244
Generalisability	21	Discuss the generalisability (external validity) of the study results	p10, lines 211-212

For peer review only - http://bmjopen.bmj.com/site/about/guidelines.xhtml

For peer review only

Other information			
Funding	22	Give the source of funding and the role of the funders for the present study and, if applicable, for the original study on which the present article is based	p13-14, lines 276-288

*Give information separately for cases and controls in case-control studies and, if applicable, for exposed and unexposed groups in cohort and cross-sectional studies.

Note: An Explanation and Elaboration article discusses each checklist item and gives methodological background and published examples of transparent reporting. The STROBE checklist is best used in conjunction with this article (freely available on the Web sites of PLoS Medicine at http://www.plosmedicine.org/, Annals of Internal Medicine at http://www.annals.org/, and Epidemiology at http://www.epidem.com/). Information on the STROBE Initiative is available at www.strobe-statement.org.

For peer review only

For peer review only - http://bmjopen.bmj.com/site/about/guidelines.xhtml
